# Transposition and duplication of MADS-domain transcription factor genes in annual and perennial *Arabis* species modulates flowering

**DOI:** 10.1073/pnas.2109204118

**Published:** 2021-09-21

**Authors:** Eva Madrid, Edouard Severing, Elisa de Ansorena, Christiane Kiefer, Luise Brand, Rafael Martinez-Gallegos, Stefan Woetzel, Ulla Kemi, Wen-Biao Jiao, Korbinian Schneeberger, George Coupland

**Affiliations:** ^a^Department of Plant Developmental Biology, Max Planck Institute for Plant Breeding Research, D-50829 Cologne, Germany

**Keywords:** *Arabis alpina*, flowering, MADS AFFECTING FLOWERING, introgression lines

## Abstract

The timing of reproduction is an adaptive trait in many organisms. In plants, the timing, duration, and intensity of flowering differ between annual and perennial species. To identify interspecies variation in these traits, we studied introgression lines derived from hybridization of annual and perennial species, *Arabis montbretiana* and *Arabis alpina*, respectively. Recombination mapping identified two tandem *A. montbretiana* genes encoding MADS-domain transcription factors that confer extreme late flowering on *A. alpina*. These genes are related to the *MADS AFFECTING FLOWERING* (*MAF*) cluster of floral repressors of other Brassicaceae species and were named *A. montbretiana* (Am) *MAF-RELATED* (*MAR*) genes. Am*MAR1* but not Am*MAR2* prevented floral induction at the shoot apex of *A. alpina*, strongly enhancing the effect of the *MAF* cluster, and *MAR1* is absent from the genomes of all *A. alpina* accessions analyzed. Exposure of plants to cold (vernalization) represses *AmMAR1* transcription and overcomes its inhibition of flowering. Assembly of the tandem arrays of *MAR* and *MAF* genes of six *A. alpina* accessions and three related species using PacBio long-sequence reads demonstrated that the *MAR*s arose within the *Arabis* genus by interchromosomal transposition of a *MAF1-like* gene followed by tandem duplication. Time-resolved comparative RNA-sequencing (RNA-seq) suggested that *AmMAR1* may be retained in *A. montbretiana* to enhance the effect of the *AmMAF* cluster and extend the duration of vernalization required for flowering. Our results demonstrate that *MAF* genes transposed independently in different Brassicaceae lineages and suggest that they were retained to modulate adaptive flowering responses that differ even among closely related species.

## Significance

Annual and perennial species differ in their timing and intensity of flowering, but the underlying mechanisms are poorly understood. We hybridized closely related annual and perennial plants and used genetics, transgenesis, and genomics to characterize differences in the activity and function of their flowering-time genes. We identify a gene encoding a transcription factor that moved between chromosomes and is retained in the annual but absent from the perennial. This gene strongly delays flowering, and we propose that it has been retained in the annual to compensate for reduced activity of closely related genes. This study highlights the value of using direct hybridization between closely related plant species to characterize functional differences in fast-evolving reproductive traits.

Annual and perennial species occur in many plant families. Annual plants and some perennials are monocarpic (flowering once in their life cycle), characterized by a massive flowering and typically produce many seeds before the whole plant senesces. By contrast, most perennials live for many years, show delayed reproduction, and are polycarpic (flowering multiple times in their life cycle) (1, 2). Therefore, annuals and perennials differ in the timing, duration, and intensity of reproduction. In both annuals and perennials, environmental cues regulate the timing of floral induction, the initial reproductive stage of higher plants, or the maturation of floral buds to ensure that flowers mature at the optimal time during the seasonal cycle to produce progeny and maximize fitness (1, 3, 4). Here, we use interspecies crosses between annual and perennial Brassicaceae species to characterize genetic differences that contribute to their distinct patterns of flowering-time control.

Annuals and perennials diverge in response to environmental pressures in relatively short evolutionary time scales, giving rise to sister annual and perennial species (4–6). Nevertheless, the genetic mechanisms underlying the divergence of these life histories are poorly understood. In *Sorghum* and *Mimulus*, a small number of genetic loci were described to have major effects in differentiating the life history of sister species (4, 7). By contrast, the separation of annual and perennial *Oryza* species was genetically complex (8). In *Mimulus*, a large inversion contributed to the divergence of annual and perennial species, which involved the differentiation of a locus with a large effect on flowering time (4, 9, 10). In the Brassicaceae, a key floral repressor gene is differentially transcribed between annuals and perennials, and this difference evolved several times to confer differences in the duration of flowering (5, 11, 12). Alterations in transcriptional patterns of key regulators have been shown to underlie rapid evolution of developmental traits in other systems and might play a broader role in divergence of annuals and perennials. In addition, gene regulatory networks can diverge rapidly through duplication or deletion of genes that encode central regulators of phenotypic traits (13, 14). Although this has not been described in the context of annual and perennial species, the reduction in genome size and genomic alterations that occurred during the evolution of annual *Arabidopsis thaliana* L. from its perennial progenitor, suggests that differences in gene content might also contribute to the evolution of annualism (15).

We have used the *Arabis* genus of the Brassicaceae as a model system to study divergence of annual and perennial species. *Arabis alpina* L. was established as a model perennial species because it is amenable to forward genetics (12) and subsequently, its sequenced genome was assembled (16, 17). Phylogenetic reconstruction showed that this species is sister to annual *Arabis montbretiana* Boiss, which enables comparisons between closely related annuals and perennials (18). Furthermore, because *A. alpina* belongs to the same family as *A. thaliana*, regulatory pathways that have been described in detail in *A. thaliana* can be relatively easily tested for their conservation or divergence in *A. alpina*. Flowering of the reference accession *A. alpina* Pajares only occurs after exposure to an extended cold period that mimics winter conditions, called vernalization. Characteristic perennial flowering patterns have been described in *A. alpina*. For example, the plant flowers after vernalization but then reverts to vegetative growth, which limits the duration of a flowering episode (12). The *PERPETUAL FLOWERING 1* (*PEP1*) gene (12), which encodes a MADS-domain transcription factor orthologous to *A. thaliana FLOWERING LOCUS C* (*FLC*) (19, 20), plays a central role in conferring these traits. In the reference accession *A. alpina* Pajares, *PEP1* represses flowering prior to vernalization, is transcriptionally repressed during cold treatment when flowering occurs, and is reactivated after exposure to cold to restrict the duration of flowering. This reactivation does not occur to the same extent in annuals such as *A. montbretiana* and *A. thaliana*, allowing them to flower indefinitely (5, 11, 19–22).

The construction of introgression lines (ILs) is a powerful genetic approach to identify genes that confer phenotypic differences between related species. In these lines, chromosomal segments from a donor parent are introduced by hybridization and backcrossing into a recipient parent. The effect of donor-parent chromosomal segments on the phenotypes of the recipient parent can then be determined. ILs can subsequently be used to rapidly develop secondary F2 populations for positional cloning of causal genes and quantitative trait loci (QTL) that underlie phenotypes of interest, including flowering time (23, 24). To facilitate the genetic study of traits modified during the divergence of annuals and perennials, an introgression line population was developed after hybridization of *A. alpina* and *A. montbretiana* (5, 11). The annual *A. montbretiana* was used as donor parent. Flowering of this species is accelerated by vernalization, but it does flower without vernalization (5, 11). Chromosomal segments from the annual donor parent were introduced into the perennial background, using the obligate vernalization requiring *A. alpina* Pajares genotype or the *pep1-1* mutant (12), and the plants screened for altered phenotypes related to flowering. Here we describe the characterization of one flowering locus identified by this approach.

We used transgenesis, long-read genomic sequencing, and RNA-sequencing (RNA-seq) to study a locus of *A. montbretiana* that strongly delays flowering of *A. alpina*. We identify a gene related to the *MADS AFFECTING FLOWERING* (*MAF*) cluster of floral repressors that has transposed to a new location in the *Arabis* genus. This transposed *MAF-RELATED* gene is present in the annuals *A. montbretiana* and *Arabis nova* subsp. *Iberica* Mart. ex Talavera, but absent from perennial *A. alpina*. We analyze the function and evolution of this gene and discuss the broader diversification of *MAF* genes in the Brassicaceae and the relevance of the transposed copy to the divergence of life history.

## Results

### Identification of *A. montbretiana MAR* Genes that Delay Flowering of *A. alpina*.

**Fig. 1.**
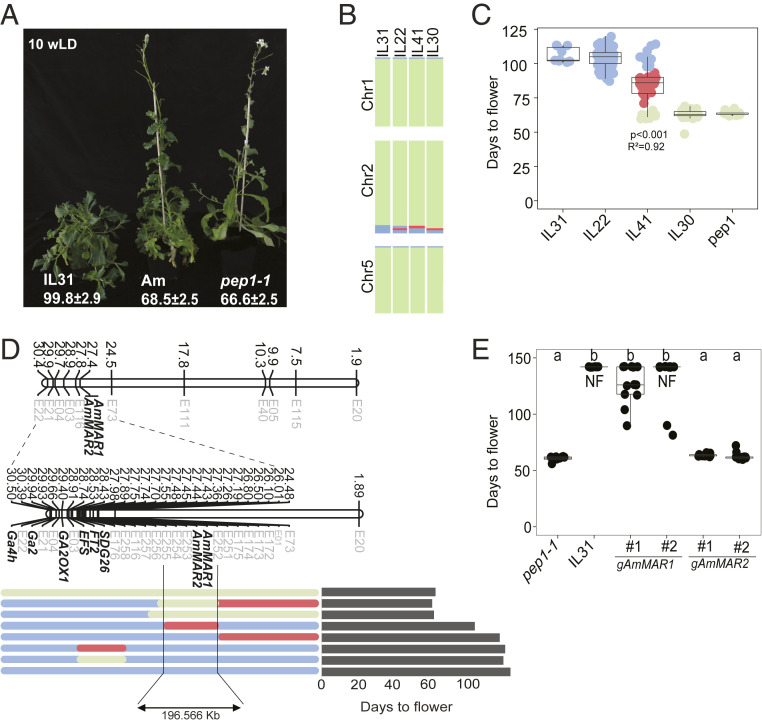
Phenotypic characterization of late-flowering plants identified from introgressing *A. montbretiana* genomic segments into *A. alpina pep1*. (*A*) Plants grown for 10 wk after germination. Days to flowering (DTF) after gemination are indicated on the *Bottom*. IL31 was later flowering than *A. montbretiana* (*Am*) and *A. alpina pep-1-1*. (*B*) Schematic representation of the genotypes of the IL used for association analysis. Different lines segregating for different fragments of chromosome 2 were chosen. Only chromosomes with introgressions from *A. montbretiana* are represented. (*C*) DTF for the ILs represented in *B*. *n* = 10 in parental lines, 70 in ILs. Only IL41 segregated for the flowering-time phenotype. The total phenotypic variation and *P* value are indicated for IL41. Individual plants are represented and color coded by the genotype of the most-associated molecular marker. (*D*) Physical map of the candidate region, showing markers (faint font) and flowering-time genes (bold font), as well as their physical positions on *A. montbretiana* chromosome 2 in megabases. The genotype of informative recombinants on chromosome 2 and the flowering time of each line is indicated on the *Right*. The candidate region that confers late flowering is located between markers E252 and E255, comprising ∼196 kb. (*E*) DTF of transgenic plants containing the genomic locus of each candidate gene in *pep1-1*. Only plants containing *AmMAR1* showed a late-flowering phenotype. *n* = 10 to 12 plants. NF, non flowering plants at the end of the experiment. Flowering phenotype is measured in days from germination to the first open flower. Letters indicate statistically significant differences determined by multiple pairwise comparisons using Tukey’s least significant difference (LSD) test (*P* ≤ 0.05). In all panels, alleles from the recurrent parent (*A. alpina pep1-1*) are colored green, alleles from the donor (*A. montbretiana*) are in blue, and heterozygous regions are marked in red.

The interspecific introgression library obtained by crossing annual *A. montbretiana* and perennial *A. alpina* was screened for plants showing altered reproductive traits. Several near-isogenic lines (NILs) containing segments of chromosome 2 of *A. montbretiana* in the *A. alpina pep1-1* background flowered much later after germination than the *A. alpina pep1-1* parent (Fig. 1*A* and *Materials and Methods*). Three lines carrying partially heterozygous introgressed segments of *A. montbretiana* chromosome 2 were selected (IL22, IL30, and IL41) for association studies (Fig. 1*B*). In addition, a line with a homozygous introgression (IL31) was used as a late-flowering control. Each line was self-fertilized and the progeny were scored for flowering time and genotyped using molecular markers designed on the basis of polymorphisms between *A. alpina* and *A. montbretiana* (Fig. 1*C* and Dataset S1). These data indicated that IL31 and IL22 were homozygous for the locus causing late flowering, whereas IL41 was heterozygous and IL30 did not contain the locus (Fig. 1*C*). Thus, the *A. montbretiana* locus that conferred late flowering was present within the heterozygous segment of introgressed DNA in IL41 that was absent in IL30 (Fig. 1*B*). Furthermore, the late-flowering phenotype segregated among the progeny of IL41 in a 1:2:1 ratio, demonstrating that it is caused by a single codominant locus (*P* < 0.01, df = 2, *χ*^2^ = 0.142, n.s. [non-significant]).

The position and size of the introgressions were defined at higher resolution by whole-genome sequencing of IL31 and IL41 (Dataset S2). Comparison of the introgressed *A. montbretiana* sequences in these lines allowed the identification of a genomic segment of about 1,617,567 bp that was associated with late flowering. This introgressed segment replaced a region of 2,339,533 bp in the *A. alpina* genome (Dataset S2). Analysis of recombinants identified in the progeny of IL41 allowed the region carrying the locus to be positioned between markers E252 and E255 on chromosome 2 of *A. montbretiana* (Fig. 1*D* and Dataset S1), a region of 196,566 bp. Comparison of the genomic sequences of this region with the orthologous region of *A. alpina* chromosome 2 revealed that 50 *A. alpina* genes were replaced by 41 genes from *A. montbretiana*. The *A. montbretiana* genes included a tandem duplication of two genes encoding MADS-domain transcription factors that were related to MADS AFFECTING FLOWERING proteins of *A. thaliana*. MAFs were previously shown to be repressors of floral transition (25, 26), but their locations on chromosome 1 (*FLOWERING LOCUS M*/*MAF1*) and chromosome 5 (*MAF2*–*MAF5*) of *A. thaliana* are not syntenic with the two genes on chromosome 2 of *A. montbretiana*. Therefore, we named the *A. montbretiana* genes *AmMAF-RELATED* (*MAR*) *1* and *AmMAR2*.

To test whether the *AmMAR* genes caused late flowering of *pep1-1*, transgenic plants were obtained that carried the genomic locus of each gene. Two independent T3 homozygous lines containing a single-locus insertion were selected for each gene construct. The transgenic *pep1-1* plants carrying the genomic locus of *AmMAR1* showed strongly delayed flowering, whereas those carrying *AmMAR2* did not (Fig. 1*E* and *SI Appendix*, Fig. S1*A*). In addition, *AmMAR1* transgenic plants flowered first on secondary shoots, and growth of the main shoot arrested in most plants. Therefore, the *A. montbretiana AmMAR1* gene confers late flowering in the *pep1-1* background, and is likely responsible for the late-flowering phenotype of the introgression lines.

### *MAR* Genes Are Inactive in *A. alpina* Pajares and Arose in the *Arabis* Genus.

**Fig. 2.**
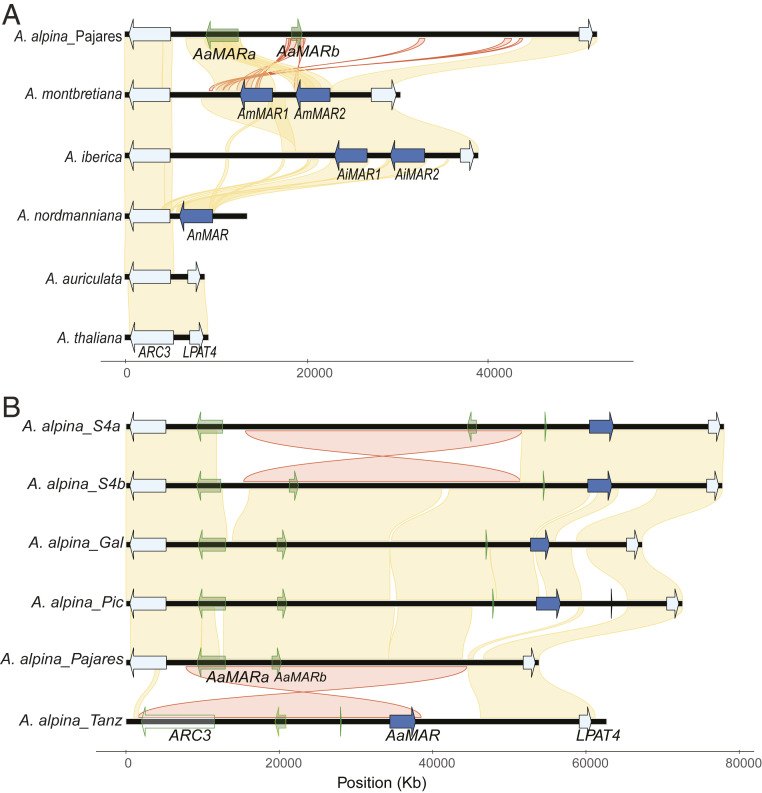
Comparison of *MAR* gene clusters among *Arabis* species and *A. alpina* accessions. (*A*) Synteny analysis between *A. thaliana* and different *Arabis* species. *MAR* genes arose after *A. auriculata* diverged in the *Arabis* clade. (*B*) Synteny analysis for *A. alpina* accessions collected across the geographical range of the species. A copy of a *MAR* gene that is predicted to be functional was identified. This copy is absent in the *A. alpina* Pajares accession. Common genes with *Arabidopsis* (*ARC3* and *LPAT4*) were taken as flanking genes for the analysis. Syntenic regions are colored in yellow and inversions in red. The accessions were collected in their natural habitats: *A. alpina*_S4a and S4b are from Scandinavia; *A. alpina*_Gal and Pic are from the French Alps; *A. alpina*_Pajares is the Spanish *A. alpina* reference accession, and *A. alpina*_Tanz is from Tanzania. In both panels, genes shown by arrows outlined or filled in with green represent predicted truncated nonfunctional proteins.

To understand why introgression of the *AmMAR* genes caused late flowering of *A. alpina pep1-1*, and to determine the evolutionary divergence of the *MAR* locus, the genome sequences of *A. montbretiana* and *A. alpina* Pajares were compared with those of the annuals *A. nova* subsp. *Iberica*, *Arabis auriculata* Lam., *Arabis nordmanniana* Rupr., and *A. thaliana*. The synteny analysis considered the common flanking genes between all species: *AtARC3* (*ACCUMULATION AND REPLICATION OF CHLOROPLAST 3*, *AT1G75010*) and *AtLPAT4* (*LYSOPHOSPHATIDYL ACYLTRANSFERASE 4*, *AT1G75020*). This analysis revealed that neither *A. thaliana* nor *A. auriculata*, which is a member of a sister clade to *A. alpina* (18), contains sequences related to *MAR1* and *MAR2* between the orthologs of *AtARC3* and *AtLPAT4* (Fig. 2*A*). In *A. montbretiana*, *AmMAR1* and *AmMAR2* are present as a tandem duplication between *AtARC3* and *AtLPAT4* (Fig. 2*A*). The same genome structure is observed in *A. nova* subsp. *Iberica*, which is closely related to *A. montbretiana*. In *A. alpina* Pajares, the reference accession for the species (16, 17), the orthologs of *AtARC3* and *AtLPAT4* are ∼40 kb apart, which includes two sequences related to *MAR* genes. However, within this interval no *MAR* genes predicted to encode full-length proteins were detected. The absence of active *MAR* genes in *A. alpina* Pajares might explain why the introgression of the active *AmMAR* genes causes late flowering of *A. alpina pep1-1*, while the presence of *MAR* pseudogenes that do not encode full-length proteins suggests that active *MAR* genes were lost in *A. alpina* Pajares following divergence from the lineage leading to *A. montbretiana*. The tetraploid species *A. nordmanniana* diverged from *A. montbretiana* after *A. auriculata* (18). Although no contig containing *ARC3* and *LPAT4* was found in the *A. nordmanniana* genome, analysis of short-read sequences showed that this species contains at least one gene closely related to *AmMAR1* and *AmMAR2* that is physically linked to *AnARC3* (Fig. 2*A*). Finally, no *MAR* genes were detected in any genome available from more distantly related Brassicaceae species. Therefore, these analyses indicate that the *MAR* genes arose after divergence of the *A. alpina/A. montbretiana/A. nordmanniana* lineage from *A. auriculata* and were subsequently lost in *A. alpina* Pajares.

The presence of two *MAR* pseudogenes in *A. alpina* Pajares (*AaMARa* and *AaMARb*) at the syntenic position to *AmMAR1/2* (Fig. 2*A*) raised the possibility that other accessions of *A. alpina* might retain active *MAR* genes. To test this, the region between *AaARC3* and *AaLPAT4* was assembled from five other accessions of *A. alpina* using PacBio reads (Fig. 2*B*). These accessions were collected across the wide geographical range of the species and included one accession from Tanzania, two from Scandinavia, and two from France, whereas Pajares was collected in northern Spain. Analysis of the genome segments of these accessions showed that they all contained one *AaMAR* gene that encoded a full-length protein (*AaMAR*). In addition, all European accessions contained both pseudogenes present in Pajares, whereas the Tanzanian accession contained a single pseudogene (Fig. 2*B*). This analysis suggests that most *A. alpina* accessions contain an active *AaMAR* gene, and that the Pajares lineage probably lost it recently.

### MAR Proteins Are Most Closely Related to the MAF1-LIKE Clade.

**Fig. 3.**
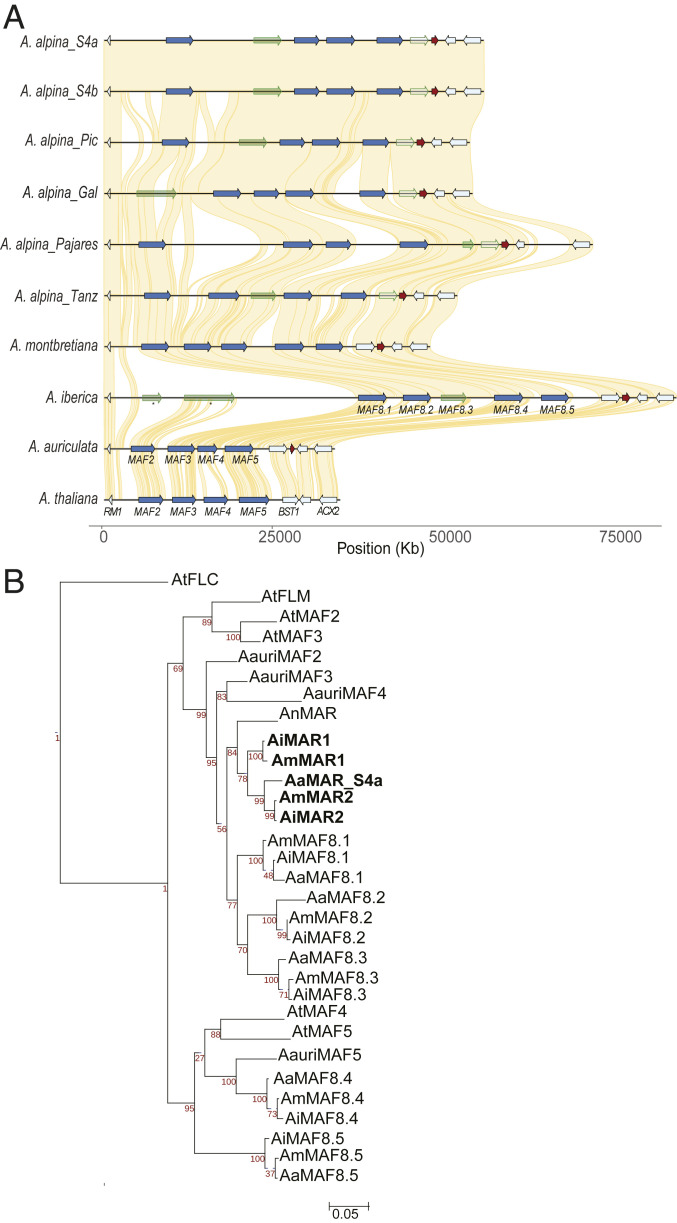
Dynamic variation in the *MAF* cluster among *A. alpina* accessions and related species. (*A*) Synteny analysis of the MAF cluster located on chromosome 8 from different accessions collected across the geographical range of *A. alpina*. Genes outlined or filled in green represent genes predicted to code nonfunctional proteins. *Arabis MAF* genes were numbered following their order on the chromosome. Common genes were taken as flanking genes for the analysis. *A. alpina* accessions were described in Fig. 2. (*B*) Maximum likelihood tree based on nucleotide alignment of the coding sequences of *FLC* clade members. Abbreviations: At (*A. thaliana*), Aauri (*A. auriculata*), An (*A. nordmanniana*), Ai (*A. nova* subsp. *Iberica*), Am (*A. montbretiana*), and Aa (*A. alpina*).

The *MAF* genes of *A. thaliana* consist of a tandem array of four genes on chromosome 5 (*MAF2*–*MAF5*) and *MAF1* (also called *FLM*) on chromosome 1. To determine whether these are conserved within the *Arabis* genus, the *MAF* clusters at the syntenic position on chromosome 8 of *A. montbretiana*, *A. nova* subsp. *Iberica*, *A. auriculata*, and six accessions of *A. alpina* were analyzed (Fig. 3*A*). *A. auriculata* contained four *MAF* genes in the cluster in a similar arrangement to that in *A. thaliana*, whereas *A. montbretiana* contained five full-length genes. *A. nova* subsp. *Iberica* and the *A. alpina* accessions all contained the five genes orthologous to those of *A. montbretiana*, but in each genome, at least one gene did not encode the full-length protein (Fig. 3*A*). Thus, the *MAF* cluster is conserved in *Arabis*, with some variation in copy number among species. No *MAF1* ortholog was detected in any *Arabis* species.

To assess the relatedness of the *MAR* genes to the *MAF* genes present in the chromosome 8 cluster, a phylogenetic tree was constructed using the coding sequences of the genes from the *MAF* cluster of *A. thaliana* and the *Arabis* species, as well as the *MAR* genes of *A. montbretiana*, *A. nova* subsp. *Iberica*, *A. nordmanniana*, and *A. alpina* (Fig. 3*B*). The *MAF* genes clustered into two major clades, one represented by *MAF1/2/3* and the other by *MAF4/5* of *A. thaliana*, as previously described (27). All of the *Arabis* species contained *MAF* genes in each clade as observed in other Brassicaceae species, suggesting that the biological functions represented by both clades are widely conserved within the family. *A. montbretiana* contained three genes in the MAF1/2/3 clade and two in the MAF4/5 clade (Fig. 3 *A* and *B*). In five of the *A. alpina* accessions, one of the genes in the *MAF1/2/3* clade, either *MAF8.1*, *MAF8.2*, or *MAF8.3*, seems to be mutated and is predicted to encode a truncated protein, whereas in *A. alpina* Pajares, *MAF8.5* from the *MAF4/5* clade was mutated (Fig. 3 *A* and *B*). *A. auriculata* contained only one gene in the *MAF4*/*5* clade, suggesting this may have duplicated in *A. montbretiana* after divergence from *A. auriculata*, and that duplication occurred independently in the *A. thaliana* lineage (Fig. 3*B*).

The *MAR* genes are located within the clade containing *A. thaliana MAF1/2/3*. Therefore, the *MAR* genes probably arose in the *Arabis* lineage after divergence of *A. auriculata* and *A. alpina*/*A. montbretiana* by transposition of a gene from the *MAF1/2/3* group (referred to below as MAF1-LIKE) from the chromosome 8 cluster to chromosome 2. The *A. nordmanniana MAR* gene is present in a separate subclade to *AmMAR1/2*, suggesting that they arose by tandem duplication after the divergence of *A. montbretiana* from the lineage leading to *A. nordmanniana*. By contrast, the active *MAR* gene present in most *A. alpina* accessions clearly associates with *AmMAR2*.

### Divergence of the *MAR* Genes from the MAF Cluster.

To understand the evolution of *MAF*/*MAR* gene sequences, the dN/dS ratio (ω) was compared among different lineages (28). Only the branch leading from *AnMAR* to the rest of the *MAR* cluster was statistically supported as showing a variable evolutionary rate compared to all other branches and showed a ω-value greater than 1 (*SI Appendix*, Fig. S2 and Dataset S3), supporting the notion that the clade containing most of the *MAR* genes is under positive selection. Furthermore, when the amino acid sites in the proteins were analyzed, the branch leading from *AnMAR* to the other *MAR* genes was again significantly different, indicating that some amino acids might be under positive selection. Among these, V43I and D61S have the highest values (Dataset S4 and *SI Appendix*, Fig. S3). These sites are within the MADS domain that is required for DNA binding. Therefore, the *MAR1* and *MAR2* genes of *A. montbretiana* and *Arabis nova *subsp. *Iberica* may have diversified from *AnMAR* by selection at specific residues.

The residues 43I and 61S are identical in AmMAR1 and AmMAR2, therefore variation at these residues does not explain the distinct functions of AmMAR1 and AmMAR2. AmMAR1 and AmMAR2 contain 20 nonsynonymous mutations, including 5 and 12 in the MADS-box and the K-box domains, respectively (*SI Appendix*, Fig. S4). The divergent region in the K domain is within the leucine-zipper motif of the second helix of the domain, and the positions of two leucine residues (position 119 in AmMAR2, and 124 in AmMAR1), previously proposed to be involved in dimerization of SEP3 (29), are altered. Thus, these changes might affect the ability of AmMAR1 and AmMAR2 to interact with partner proteins and explain the apparent specificity of AmMAR1 in delaying flowering.

### *MAR1* Represses Flowering of the Primary Inflorescence, Reduces Shoot Elongation, and Confers a Vernalization Response.

**Fig. 4.**
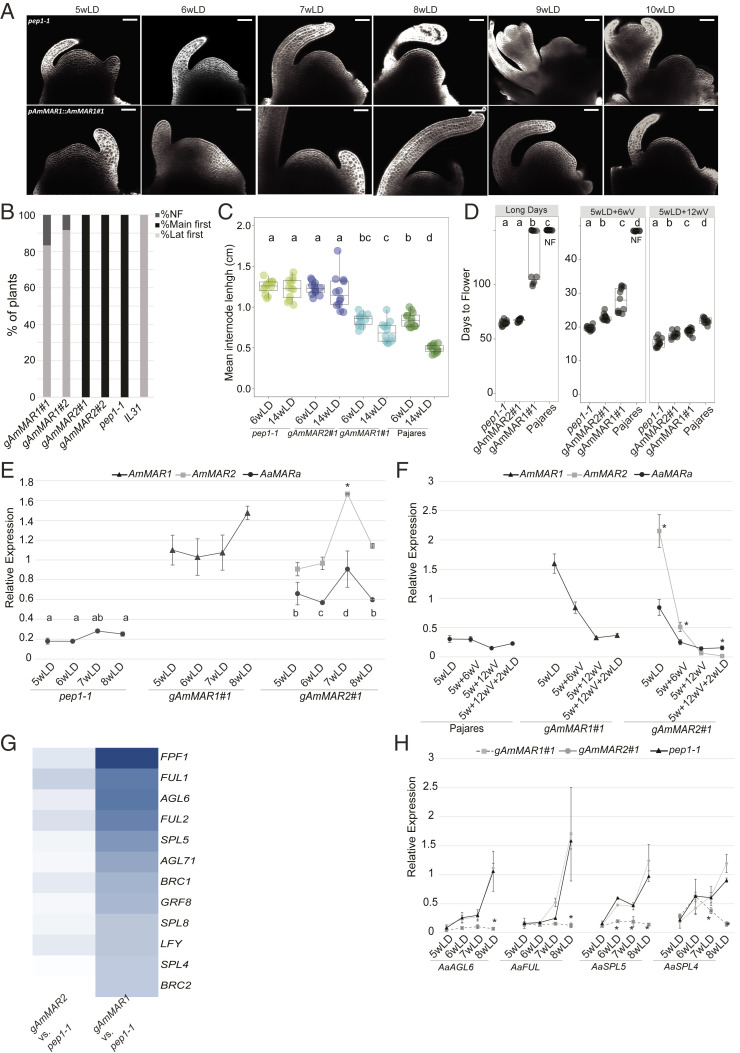
*AmMAR1* represses floral transition of *pep1-1* mutants under LD. (*A*) Meristem morphology in *pep1-1* and g*AmMAR1::gAmMAR1* at different time points in long-day conditions. Transgenic plants carrying *AmMAR1* are not induced to flower after 10 wk in long days, when *pep1-1* mutants have well-developed floral buds. (Scale bar: 50 µm.) (*B*) Percentage of plants flowering first on the main and lateral shoots. Plants carrying *AmMAR1* flowered mainly on secondary lateral shoots, whereas *pep1-1* and *AmMAR2* plants flowered first on the main shoot. Lat. first, first flower formed on lateral shoot; main first, first flower formed on main shoot; NF, not flowering. (*C*) Mean internode length for Pajares and late-flowering transgenic plants carrying *AmMAR1*, compared with that of *pep1-1* and *pAmMAR2::gAmMAR2* after 6 and 14 wk in long days. Late-flowering plants had shorter internodes. The error bars represent the SD; *n* = 12. (*D*) Comparison of the flowering time of transgenic *MAR* lines, perennial *A. alpina* Pajares, and *pep1-1* without vernalization in long days and after two vernalization periods. *pAmMAR1::gAmMAR1* flowers late without vernalization, the reference Pajares never flowers without vernalization, and *pep1-1* flowers perpetually. The differences in flowering time are strongly reduced by vernalization and are smaller after vernalization for 12 wk. (*E*) Levels of *AaMARa*, *AmMAR1*, and *AmMAR2* mRNA in the main inflorescence apex of *AmMAR1* and *AmMAR2* transgenic lines and *pep1-1* growing for 8 wk in long days. (*F*) The level of mRNA of *PEP1*, *AaMARa*, *AmMAR1*, and *AmMAR2* in apices without vernalization or on exposure to different vernalization periods. Expression of *AmMAR* genes is strongly repressed after 6 wk of vernalization (*n* = 12). (*G*) Heat map of DEGs for flowering time according to the log_2_-fold change (log_2_FC) values for *pAmMAR1::gAmMAR1* and *pAmMAR2::gAmMAR2*. All DEGs are listed in Dataset S3. (*H*) Expression level of selected DEGs in shoot apices after growth for 5 to 8 wk in long days. The data represent the means of two biological replicates, and error bars represent the SD. Asterisks above or below the datapoints indicate significant differences determined by multiple pairwise comparisons using Tukey’s honestly significant difference (HSD) test (*P* ≤ 0.05). qPCR data are the mean of two biological replicates, and error bars represent the SD. Asterisks above the datapoints indicate significant differences determined by multiple pairwise comparisons using Tukey’s HSD test (*P* ≤ 0.05).

The role of AmMAR1 in the repression of floral transition was characterized in more detail. Microscopic analysis of the shoot apical meristem demonstrated that *pep1-1* formed well-developed floral primordia by 9 wk after germination, whereas the morphology of the shoot apex of *pAmMAR1::gAmMAR1* plants remained vegetative 10 wk after germination (Fig. 4*A*). In *pep1-1* and *pAmMAR2::gAmMAR2* transgenic plants, flowers always appeared first at the shoot apex, whereas most *pAmMAR1::gAmMAR1* plants produced flowers and seeds from secondary inflorescences, and no flowers were visible at the shoot apex (Fig. 4*B* and *SI Appendix*, Fig. S1*B*). Therefore, the repression of flowering caused by *AmMAR1* appears to be stronger at the shoot apical meristem than on lateral branches. In addition to delaying flowering, *AmMAR1* reduced plant height and the length of internodes in the primary shoot (Fig. 4*C* and *SI Appendix*, Fig. S1*C*). Internode length of wild-type *A. alpina* Pajares plants was also shorter than that of *pep1-1* mutants (Fig. 4*C*) (30), indicating that AmMAR1 and PEP1 have similar effects on plant height as well as flowering time.

The effect of *MAF* genes on flowering of *A. thaliana* can be overcome by vernalization (26); therefore, the flowering time of the *MAR* transgenic plants was tested after vernalization. Five-week-old *pAmMAR1::gAmMAR1* plants vernalized for only 6 wk flowered within 32 d after vernalization, whereas the Pajares reference accession did not flower (Fig. 4*D*). After this short vernalization treatment, *pAmMAR1::gAmMAR1* all flowered at the shoot apex and on lateral branches, although *pAmMAR1::gAmMAR1* flowered later than *pAmMAR2::gAmMAR2* and *pep1-1* plants and *pAmMAR2::gAmMAR2* flowered slightly later than *pep1-1* (Fig. 4*D*). After 12 wk of vernalization, Pajares also flowered and the differences in flowering among genotypes were smaller (Fig. 4*D*). Thus, the strong repression of flowering caused by *pAmMAR1::gAmMAR1* can be largely overcome by short vernalization treatments of 6 wk.

To understand further the function of *AmMAR1* and *AmMAR2*, their mRNA levels were analyzed in different tissues and at various times after germination. Each mRNA was highly expressed in the corresponding transgenic plant in all tissues and time points tested, with the lowest expression in cotyledons (*SI Appendix*, Fig. S5*A*). In *pep1-1*, the *AmMAR1* primers amplified the transcript formed from one of the *A. alpina* Pajares pseudogenes, and the abundance of this transcript was greater in *pAmMAR2::gAmMAR2* transgenic plants, suggesting that AmMAR2 can directly or indirectly activate this gene (*SI Appendix*, Fig. S5*A*). Apices of the transgenic plants and *pep1-1* were then analyzed between 5 and 8 wk after germination, when the plants underwent floral induction. The mRNA level of *AmMAR1* and *AmMAR2* remained high throughout the time course (Fig. 4*E*), demonstrating that *AmMAR2* expression is not reduced during floral induction of *pAmMAR2::gAmMAR2* plants, and that *AmMAR1* mRNA is present in apices of *pAmMAR1::gAmMAR1* plants when their phenotypes start to diverge from those of *pep1-1*. In the first few lateral shoots of the primary stems of the transgenic plants, high levels of *AmMAR1* and *AmMAR2* mRNAs were also detected, similar to those found in the primary shoot apex at these time points (*SI Appendix*, Fig. S5*B*).

The delay in flowering of *pAmMAR1::gAmMAR1* compared with *pep1-1* was overcome by vernalization (Fig. 4*D*); therefore, the mRNA levels of *AmMAR1* and *AmMAR2* were quantified in apices before and after exposure to 6 and 12 wk of vernalization (Fig. 4*F*). The expression of both genes was strongly repressed by the end of the 12-wk vernalization treatment (Fig. 4*F*) and remained low 2 wk after vernalization. After 6 wk of vernalization, *AmMAR1* and *AmMAR2* mRNA abundance was strongly reduced (Fig. 4*F*), indicating that the early flowering of *pAmMAR1::gAmMAR1* after vernalization is probably due to repression of *AmMAR1* transcription.

### Effects of *AmMAR1* on Gene Expression.

To determine the effect of *AmMAR1* on gene expression at the shoot apex, the transcriptomes of both transgenic lines and *pep1-1* were determined by RNA-sEq 6 wk after germination. At this time point, the apex of the *pep1-1* mutant was at an early stage of floral transition, whereas *pAmMAR1::gAmMAR1* transgenic plants remained vegetative (Fig. 4*A*). As expected, more differentially expressed genes (DEGs) were found between *pAmMAR1::gAmMAR1* and *pep1-1* than between *pAmMAR2::gAmMAR2* and *pep1-1*, and only 17 were common between both comparisons (*SI Appendix*, Fig. S6*A* and Dataset S5). We performed Gene Ontology (GO) enrichment analysis for the 167 DEGs in *pAmMAR1::gAmMAR1*. GO terms related to meristem maintenance, positive regulation of flower development, and regulation of the vegetative phase, were significantly enriched in this analysis (*SI Appendix*, Fig. S6*B*). Notably, expression of most DEGs was reduced in *pAmMAR1::gAmMAR1* compared with *pep1-1* (Fig. 4*G*). One of the most down-regulated genes in *pAmMAR1::gAmMAR1* was *FLOWERING PROMOTING FACTOR 1* (*AaFPF1*), which in *A. thaliana* is up-regulated during floral transition and causes early flowering when overexpressed from a heterologous promoter (31). Furthermore, the expression of several genes encoding orthologs of transcription factors with established roles in floral induction and floral meristem identity of *A. thaliana* was also reduced in *pAmMAR1::gAmMAR1* (Dataset S5). These data indicate that MAR1 represses the expression of many genes involved in the early stages of floral transition.

To test differences in the dynamics of flowering-time gene expression, RNA was harvested from apices of *pAmMAR1::gAmMAR1*, *pAmMAR2::gAmMAR2*, and *pep1-1* plants grown for 5 to 8 wk under long days. Four DEGs identified by RNA-seq were analyzed by qRT-PCR in all three genotypes across the time course in main and lateral shoots (Fig. 4*H* and *SI Appendix*, Fig. S6*C*). The mRNA abundance of *AaFUL*, *AaAGL6*, *AaSPL4*, and *AaSPL5* all increased during the time course in *pAmMAR2::gAmMAR2* and *pep1-1*, but remained at low levels in *pAmMAR1::gAmMAR1* (Fig. 4*H*). In lateral shoots, a slight increase in *AaAGL6*, *AaSPL4*, and *AaSPL5* was observed in the final time point (*SI Appendix*, Fig. S6*C*). These data support the conclusion that AmMAR1 strongly blocks the early stages of the floral transition at the primary shoot apex.

### Comparative Analysis of the Responses of *MAF* and *MAR* Gene Expression to Vernalization in *A. thaliana*, *A. montbretiana*, and *A. alpina*.

**Fig. 5.**
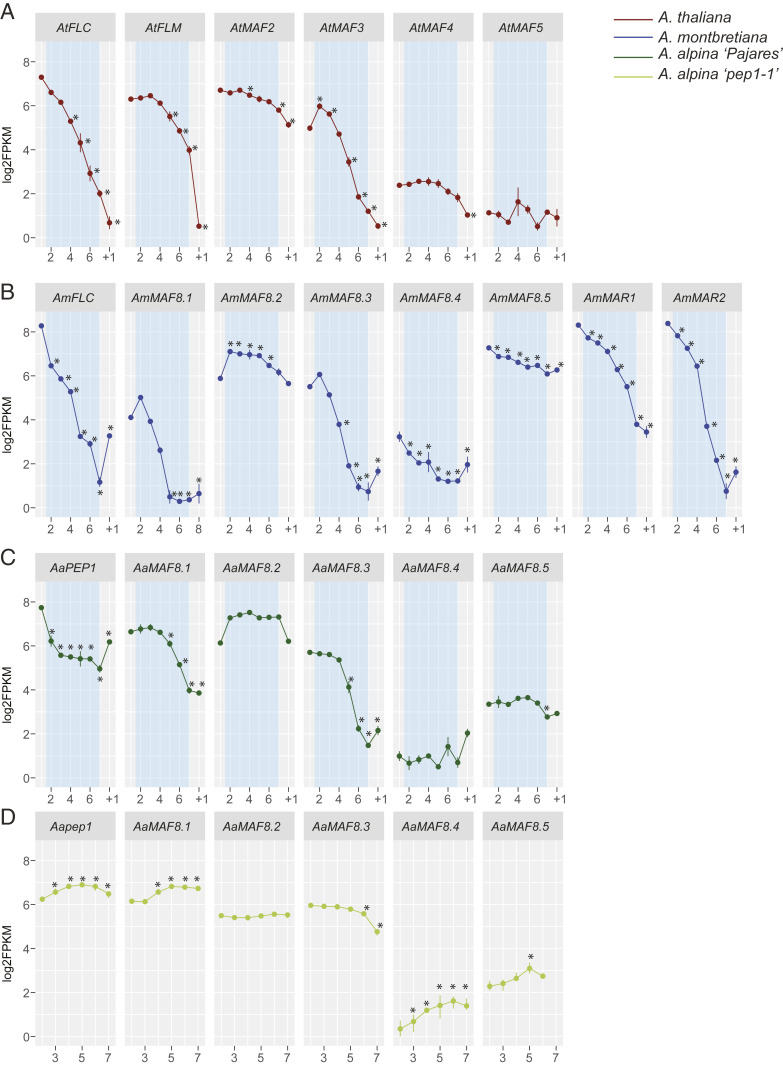
The transcriptional dynamics in different genotypes of *MAF* and *MAR* gene expression in response to vernalization. Log_2_ FPKM (fragments per kilobase of transcript per million mapped reads) for orthologs of *FLC*, *MAF*, and *MAR* genes before, during, and after vernalization for *A. thaliana* (*A*), *A. montbretiana* (*B*), and *A. alpina* Pajares (*C*), and *A. alpina pep1-1* growth in long days with no vernalization harvested weekly from 2 to 7 wk after germination (*D*). For *A*–*C*, shoot apices were harvested before vernalization (2 wk for *Arabidopsis* and *A. montbretiana* or 6 wk for *A. alpina* Pajares), during vernalization treatment (2, 4, 6, 8, 10, and 12 wk), and 1 wk after plants were returned to warm temperatures. The blue box indicates the duration of vernalization. The genes are ordered according to their chromosomal position and not their evolutionary relationships. Asterisks indicate significant differences calculated using DESeq2 of the fold changes versus the first time point. *AaMARa*, which does not encode an active protein, is not represented.

In *A. thaliana*, *MAF* genes extend the duration of vernalization required for flowering and differ in their rate of repression by vernalization (25, 26, 32). However, the expression of all *MAF* genes during vernalization has not been quantitatively tested and the extent to which their responses to vernalization are conserved in different Brassicaceae species is unknown. To address these issues, RNA-seq was used to determine the mRNA levels of *FLC*, *MAF*, and *MAR* genes during vernalization in *A. thaliana*, *A. montbretiana*, and *A. alpina* Pajares, as well as in *pep1-1* mutants not exposed to vernalization (Fig. 5 and Dataset S6).

In *A. thaliana*, the mRNAs of *AtFLC* and *AtMAF1/2/3* were present at high levels prior to vernalization, and the mRNA abundance of *AtFLC*, *AtMAF1* (*FLM*), and *AtMAF3* decreased rapidly in cold, whereas that of *AtMAF2* decreased more slowly. By contrast, *AtMAF4/5* mRNAs were expressed at lower levels, and the *AtMAF4* mRNA level was reduced slowly by cold (Fig. 5*A*). These results are in broad agreement with previous reports (25, 26). Remarkably similar patterns were detected in *A. alpina* and *A. montbretiana*, although with significant differences (Fig. 5 *B* and *C*). In *A. montbretiana*, expression of *AmFLC*, two of the *AmMAF1-LIKE* genes (*AmMAF8.1* and *AmMAF8.3*), and *AmMAR2* was rapidly reduced during vernalization, whereas expression of *AmMAR1* and *AmMAF8.2* fell more slowly (Fig. 5*B*). The mRNAs of the *MAF4-LIKE* genes also reduced only slowly during vernalization, similar to that of the *A. thaliana* genes in this group. In *A. alpina*, the *PEP1* and *AaMAF1-LIKE* genes were generally less responsive to vernalization (Fig. 5*C*). During vernalization, the abundance of *PEP1* and *AaMAF8.1* mRNA was reduced slowly and was still relatively high after the 12-wk vernalization treatment (Fig. 5*C*). In the *pep1-1* mutant, which was not exposed to vernalization, the expression of *AaMAF8.1*, *-8.2*, and -*8.3* was almost unchanged through the time course, showing that the repression of these genes in *A. alpina* Pajares during vernalization is due to exposure to cold (Fig. 5*D*). Collectively, these results indicate that in different lineages of the Brassicaceae, *FLC* and most of the *MAF1*-related genes are highly expressed prior to vernalization and repressed during vernalization, although the rate of reduction may differ between species, as observed in the relatively slow rate of repression of these genes in *A. alpina*. In addition, the transcriptional patterns of the *AmMAR* genes are most similar to those of the *MAF1-LIKE* genes, consistent with their position in the phylogeny, but *AmMAR1* is expressed more highly than the *AmMAF* genes prior to vernalization and is repressed by vernalization at a slower rate than *AmFLC* or *AmMAR2*.

## Discussion

We used interspecies hybridization, long-read genomic sequencing, and RNA-seq to identify a tandem array of genes encoding MAR MADS-domain transcription factors that arose in the *Arabis* genus, strongly repress flowering at the shoot apex, and differ in their arrangement among closely related species as well as among accessions of *A. alpina*. Our data emphasize the importance of transposed *MAF* gene copies in flowering-time regulation and show that these have arisen independently in different lineages of the Brassicaceae. We discuss our data in the context of duplication and retention of plant genes and their significance in life-history divergence.

### Tandem Duplication and Transposition of *MAF* Genes in the Brassicaceae.

Gene duplication contributes to the appearance of novel traits during plant evolution (33–35). Duplicates arise by whole-genome duplication or different mechanisms of single-gene duplications such as transposition or unequal crossover at meiosis to create a tandem duplication. Tandem duplicates have often been misannotated as single-copy genes because of the difficulty of assembling them from short-read sequencing data (33). The PacBio long-read sequences used here enabled the assembly of the tandem arrays of *MAF* and *MAR* genes from several *Arabis* species. The *MAF* gene cluster occurs at a syntenic position to the one in *A. thaliana* (26) and *Brassica rapa* (27), whereas the *MAR* cluster arose by transposition within *Arabis*.

Ancestral genome and phylogenetic reconstructions indicated that the *MAF* gene cluster arose from tandem ancestral *MAF1-LIKE* and *MAF4-LIKE* genes that were present in the stem group of the core Brassicaceae (27). In *A. thaliana,* four *MAF* genes are present in the cluster (26, 36): two derived from the ancestral *MAF1-LIKE* gene and two from the *MAF4-LIKE* gene, and these duplications are proposed to have occurred in lineage I leading to *A. thaliana* (27). Among accessions of *A. thaliana*, naturally occurring mutations in this cluster, particularly in *MAF2* and *MAF3*, cause earlier flowering (36, 37). In *Arabis*, which is in lineage IV of the Brassicaceae and diverged from the lineage leading to *A. thaliana* around 23 million years ago (38, 39), the *MAF* cluster has amplified independently. *A. auriculata* and *A. alpina* Pajares, the reference accession, each contain three genes derived from the ancestral *MAF1-LIKE* gene, and one derived from the ancestral *MAF4-LIKE* gene, whereas *A. montbretiana* contains an extra *MAF4-LIKE* gene that is also present in other accessions of *A. alpina*. In *Brassica* species in lineage II of the Brassicaceae, descendants of *MAF1-LIKE* and *MAF4-LIKE* are also present in the *MAF* gene cluster (27, 40). Therefore, amplification and loss of *MAF* genes within syntenic tandem arrays have occurred frequently and independently in different lineages of the Brassicaceae.

In addition to the tandem array of *MAF* genes, transposed copies occur on other chromosomes. In *A. thaliana*, *AtMAF1* (also called *AtFLM*) is located on a different chromosome from the *AtMAF* cluster and confers late flowering (41, 42). *AtMAF1* is proposed to have arisen by transposition of an ancestral *MAF1-LIKE* gene out of the *AtMAF* cluster. No ortholog of *AtMAF1* was detected in the genomes of *Brassica* species, suggesting that transposition occurred in lineage I (27, 40). Similarly, in *Arabis* species, an ortholog of *AtMAF1* is absent, but independent transposition of a *MAF1-LIKE* gene occurred, generating the *MAR* genes at a position that is not syntenic with *AtMAF1*. The transposed *MAR* gene then duplicated, creating a tandem array of *AmMAR1* and *AmMAR2* in *A. montbretiana* and *A. nova spp. Iberica*, although only *AaMAR2* is present in *A. alpina*. The presence of transposed copies in *Brassica* is more difficult to determine because of the genome triplication and reassortment that occurred in that lineage. Nevertheless, our analysis of the *Arabis* genus clearly demonstrates that in addition to amplification of the syntenic *MAF* cluster, recent, independent transposition events have generated additional copies of *MAF1-LIKE* genes at different locations in separate Brassicaceae lineages.

### Functions of *MAF* Genes and the Retention of Duplicate Copies.

Single-gene duplication occurs frequently through unequal crossover or transposition (33, 43), but most duplicates are subsequently lost and are either deleted from the genome or become pseudogenes (44, 45). The syntenic *MAF* clusters of all species tested in the core group of the Brassicaceae contain descendants of both the ancestral *MAF1-LIKE* and *MAF4-LIKE* genes, suggesting that these two gene lineages have distinct functions and are retained by selection (26, 40). The *MAF1-LIKE* genes are present in multiple copies in all genomes tested. Several explanations have been proposed for the retention of gene copies, including gene dosage, subfunctionalization, and neofunctionalization (33, 46). In *A. thaliana*, genetic analysis demonstrated that all of the members of the *MAF1-LIKE* group, *AtMAF1*, *AtMAF2*, and *AtMAF3*, as well as *AtMAF4* in the *MAF4-LIKE* group, delay flowering, because mutation of each gene causes earlier flowering (25, 26). Notably, the single mutation with the strongest effect is *maf1*, in which the transposed copy is inactivated and the genes in the *MAF* cluster are still active (25, 41). Moreover, each *maf* mutation has a stronger early-flowering phenotype at 16 °C than at 23 °C, suggesting that the genes are particularly important in delaying flowering at low temperatures (25, 41). Similarly, the *MAF* genes were proposed to enhance the duration of vernalization required to promote flowering, because *maf* mutants flowered after exposure to shorter vernalization treatments (25, 26). Combining mutations in *AtMAF1*, *AtMAF2*, and the related floral repressor *AtFLC* caused an extreme early-flowering phenotype even at 16 °C (25). Thus, the *AtMAF* genes are partially redundant with each other and with *AtFLC*, suggesting that they have an additive effect on floral repression. However, they probably also have qualitatively distinct effects. For example, in contrast to transcription of *AtFLC*, that of *AtMAF2* is repressed slowly or not at all by vernalization (26), and this may confer its capacity to extend the duration of vernalization required for flowering. Moreover, *AtMAF1* and *AtMAF2* transcripts are differentially spliced at higher temperatures, reducing the activity of the gene and allowing earlier flowering (32, 47, 48). Overall, the *AtMAF* genes play important roles in delaying and modulating flowering time in response to changes in temperature, in the context of vernalization or ambient temperature changes. Therefore, the retention of *MAF* genes after duplication might be a consequence of selection for altered flowering time in response to changes in environmental temperatures (27).

In *Arabis*, *AmMAR1* confers extreme late flowering in *pep1-1* mutants that also contain the full *AaMAF* cluster. Therefore, the transposed copy delays flowering more strongly than the ancestral *MAF* cluster, as described for *AtMAF1* in an *A. thaliana flc* mutant (25). The *AaMAF* cluster alone has a relatively weak effect on flowering time in the absence of *PEP1* and *MAR1*, as shown by the early-flowering phenotype of the *pep1-1* mutant and our observation in RNA-seq analysis that the whole *AaMAF* cluster is expressed in apices of *pep1-1* mutants (Fig. 5*D*).

Nonsynonymous amino acid changes and altered transcriptional patterns may contribute to the stronger delay of flowering caused by *AmMAR1* in *A. alpina* than the ancestral *AaMAF* cluster. Genome-wide analyses in rice and *A. thaliana* previously demonstrated that dispersed gene duplicates, such as those generated by transposition, tend to be more diverged in gene expression pattern than tandem duplicates (49, 50). Consistent with these observations, in our RNA-seq analysis *AmMAR1* transcripts were more abundant prior to vernalization than those of any of the other *AmMAF* genes and this increased expression may contribute to the stronger phenotypic effect of the transposed copy. However, we also found that the rate of nonsynonymous changes in the *MAR* clade after divergence from *AnMAR* was higher than in the ancestral *MAF* clade, consistent with accelerated protein evolution after transposition. Particularly, the described changes in the K domain between MAR1 and MAR2 may alter protein interactions and enhance the effect of MAR1 on flowering time.

Transgenic *A. alpina pep1-1* mutants carrying *AmMAR2* did not flower later than the parental plants without vernalization; however, these plants did flower slightly later than *pep1-1* mutants after short vernalization periods. Therefore, MAR2 may have a weaker but significant role in modulating flowering time, comparable to genes in the *AtMAF* cluster of *A. thaliana*. *MAR2* is also present in all *A. alpina* accessions (except Pajares), as well as in *A. montbretiana* and *A. nova* subsp. *Iberica*, suggesting that it has been retained by selection during the divergence and diversification of these species.

### *MAF* Genes and the Divergence of Annual and Perennial Life History.

Flowering is generally more strongly repressed in perennial Brassicaceae species than in their annual counterparts; therefore, it was unexpected that annual *A. montbretiana* and *A. nova* subsp. *Iberica* have retained *MAR1*, a strong repressor of flowering, whereas the gene is absent in all accessions of perennial *A. alpina* tested. Whereas *AmMAR1* effectively prevents flowering of *pep1-1* on the main shoot, *A. montbretiana* flowers in the absence of vernalization when the *AmMAR*, *AmMAF*, and *AmFLC* genes would be expected to be expressed. How the effect of these repressors on floral transition is overcome in *A. montbretiana* is unknown, but probably other flowering pathways act independently of these repressors to bypass their effect on gene expression and floral transition. Such interactions among pathways have been extensively analyzed in *A. thaliana* (51). Extensive genetic analysis in *A. montbretiana* will be required to determine the effect of *AmMAR1* loss of function, and this will require the development of transformation protocols and reverse genetics for this species.

The RNA-seq analysis suggests the selection pressure to retain *MAR1* in the annuals may be explained by compensation among other members of the *MAF* family. In *A. montbretiana*, the *MAF1-LIKE* genes in the *MAF* cluster that are repressed by vernalization, *AmMAF8.1* and *AmMAF8.3*, are expressed at relatively low levels prior to vernalization and are rapidly repressed during vernalization. By contrast, *AmMAR1* is expressed four- to fivefold higher than these genes prior to vernalization and its expression is reduced more slowly during vernalization. Therefore, *AmMAR1* may be retained in *A. montbretiana* to compensate for the lower level of expression of the related genes in the *MAF* cluster. Similarly, *AtMAF2* was proposed to extend the duration of vernalization required for flowering of *A. thaliana* (25, 26). The requirement for MAF1 in *A. alpina* may be weaker because *AaMAF8.1* is expressed at a higher level than its ortholog in *A. montbretiana* prior to vernalization, and its repression during vernalization is much more gradual. Applying CRISPR-Cas9 for reverse genetic analysis to test the contribution of *MAF* genes to flowering time and vernalization response in the *Arabis* species would help resolve the contributions of individual genes.

The *FLC* ortholog *PEP1* is related to the *MAF* genes and plays a central role in the perennial life cycle of *A. alpina* by restricting floral induction to a short time period at the end of vernalization (12, 52). Our comparative RNA-seq analysis showed that the *FLC* orthologs are much more rapidly repressed by cold in the annuals *A. thaliana* and *A. montbretiana* than in perennial *A. alpina*, and that in the latter, *PEP1* is still significantly expressed after 12 wk of vernalization. The duration of vernalization response in *A. alpina* may therefore be strongly determined by *PEP1* expression, and this may ensure that the plant requires longer vernalization treatments for flowering to occur than in the annuals. This suggestion is consistent with previous observations that *FLC* alleles of *A. thaliana* that differ in the rate of repression by vernalization determine the duration of vernalization required for flowering (53), and that short vernalization treatments do not allow full inflorescence development in *A. alpina* (22). Our analysis of the *FLC* and *MAF* orthologs of these annual and perennial species, therefore, suggests that the selection pressure to retain *MAR1* may be stronger in the annuals than in the perennial, because of differences in the patterns of expression of their respective *FLC* orthologs and *MAF* gene clusters. In this case, because *PEP1* and vernalization response play a central role in the perennial life history of *A. alpina*, the absence of *MAR1* in this species would be an indirect consequence of its perennial life history.

## Materials and Methods

### Plant Material and Growth Conditions.

The perennial *A. alpina* reference accession Pajares, the *pep1-1* mutant, and annual *A. montbretiana* (accession BM7968, provided by Birol Mutlu, Turkey) were used as parent lines to generate the populations used in this study (12, 18). Parental *A. montbretiana* flowers without vernalization around 68 d after germination, but contains an active *FLC* ortholog, and its flowering time is accelerated by vernalization treatment (5, 11). The *A. alpina* Pajares parent shows an obligate vernalization requirement, while the *pep1-1* mutant flowers without vernalization (12). An F1 population was obtained for the cross *A. montbretiana* × *A. alpina* Pajares and was then backcrossed to both Pajares and *pep1-1*. Up to 35 plants for each family were self-fertilized to generate BC1S1 seeds and genotyped by genotyping by sequencing (GBS). Using this information, 44 lines with introgressed fragments of *A. montbretiana* fixed in the *pep1-1* background were obtained.

For flowering-time experiments, seeds were stratified in darkness for 3 to 5 d at 4 °C. Plants were then grown in the glasshouse under long days (LDs) (16 h light:8 h dark) at a light intensity of 200 to 500 µmol m^−2^ s^−1^ at 22 °C. Vernalization was performed in a short days (SDs) growth chamber at 4 °C and a light intensity of 14 µmol m^−2^ s^−1^. Days to flower (DTF) was measured for each genotype as days to the first open flower from germination. All experiments were performed with at least 12 plants.

### Marker Development, Genotyping, and Whole-Genome Sequencing.

To estimate and characterize the annual introgressions, lines of interest were subjected to whole-genome sequencing using Illumina HiSeq3000 with150 bp (paired-end reads, Project no. PRJNA532504, biosamples SAMN18581157 [IL31] and SAMN18581158 [IL41]). The cleaned reads were mapped to the pooled genomes of *A. alpina* (Pajares) V5.1 (16) and *A. montbretiana* V3.1 using BWA (54) and the number of read pairs that mapped to each annotated gene was determined. The counts were normalized based on the total number of reads mapped and the length of the gene, to obtain fragments per kilobase of transcript per million mapped reads (FPKM) values. To determine which regions of the *A. montbretiana* genome were introgressed into *A. alpina*, we first identified syntenic blocks between the two genomes by performing whole-proteome Blast (55) searches (e value) using the output for the DAGCHAINER program (56). Finally, the FPKM ratio between the FPKM of the matching genes (Aa/Am) in the syntenic blocks was used to determine whether the gene was homozygous *A. alpina* (2), homozygous *A. montbretiana* (0.5), or heterozygous.

Once introgressions were defined, plants were genotyped using primer pairs specifically designed within the introgressed intervals (Dataset S1).

### RNA Extraction and qRT-PCR Analysis.

RNA was extracted using the RNeasy Plant Mini Kit (Qiagen) and treated with DNA-free DNase (Ambion). Total RNA (1.5 μg) was used to synthesize cDNA with SuperScript IV Reverse Transcriptase (Invitrogen) with oligo(dT)18 as a primer. Transcript levels were quantified by quantitative PCR in a LightCycler 480 (Roche) and iQ SYBR Green Supermix detection system (Bio-Rad). Each data point was derived from two biological and three technical replicates and represents the mean ± SD. LightCycler melting curves were obtained for the reactions, revealing single peak melting curves for most amplification products. *AaRAN3* and *AaUBI* were used for normalization (57). The sequences of the primers used in this work are listed in Dataset S1.

### RNA-Seq Analysis.

RNA-seq studies were used to study 1) differential gene expression in apices of 6-wk-old *pep1-1* plants carrying *AmMAR1 or AmMAR2* transgenes (PRJNA730091); 2) the expression of *MAF* genes in shoot apices before vernalization (2 wk for *Arabidopsis* and *A. montbretiana* or 6 wk for *A. alpina* Pajares), during vernalization treatment (2, 4, 6, 8, 10, and 12 wk), and 1 wk after plants were returned to warm temperatures (PRJNA730701); and 3) expression of *MAF* genes in the absence of *PEP1* in shoot apices of *pep1-1* plants at 2, 3, 4, 5, 6, and 7 wk after germination in long days (PRJNA728651). In all RNA-seq experiments, apices of 12 to 20 plants were harvested at each time point for each biological replicate (three for time frames 1 and 3 [above]; two for time frame 2 [above]).

RNA was isolated as described above and RNA integrity was confirmed on an Agilent BioAnalyzer. Library preparation and sequencing were performed at the Max Planck Genome Center Cologne, Germany (https://mpgc.mpipz.mpg.de/home/). Poly(A) RNA was isolated from 1 µg of total RNA using NEBNext Poly(A) mRNA Magnetic Isolation Module (New England Biolabs) and used for library construction with NEBNext Ultra Directional RNA Library Prep Kit for Illumina (New England Biolabs). RNA-seq was performed on an Illumina HiSEq. 3000 system with 150-bp single-read lengths.

RNA-seq–based expression levels were quantified using Salmon (58). Batch effects were identified in the vernalization dataset and corrected using the sva R package (59). FPKM normalization for visualization was obtained using the fpkm function of the DESeq2 package (60), which uses robust scaling factors rather than the total fragment depth. Differential expression analysis was performed with DESeq2.

### Synteny and Phylogenetic Analysis.

For synteny analysis, nucleotide alignments between each sequence pair compared were generated using lastal (61). The initial alignment blocks were further processed using SyRI (62), resulting in a minimum set of alignments reflecting colinearity or rearrangements between the sequences.

To generate maximum likelihood (ML) trees, initial amino acid alignments generated with MUSCLE were processed by removing poorly conserved regions using trimAl (-automated1) (63, 64). The trimmed amino acid alignments were converted to codon alignments by replacing each amino acid with its corresponding codon from the original nucleotide sequence and multiplying gaps by 3. jModelTest (65) was used to determine the most appropriate substitution model for subsequent maximum likelihood tree reconstruction based on the Bayesian information criterion (BIC). The final ML tree was generated using PhyML (66) with parameters suggested by jModelTest, including 100 bootstrapping replicates. Phylogenetic trees were rendered after midpoint rooting using the ete3 python package (67).

For dN/dS analysis, the original MUSCLE alignment was trimmed by removing positions with more than 20% gaps and then converted into a codon alignment suitable for PAML (28). Parameter settings for the branch and branch site tests were set according to the PAML manual.

## Supplementary Material

Supplementary File

Supplementary File

Supplementary File

Supplementary File

Supplementary File

Supplementary File

Supplementary File

## Data Availability

Sequencing raw data have been deposited in NCBI (PRJNA728651: Whole-genome sequencing of IL PRJNA728651: RNA-seq time series of *A. alpina* pep1-1 apices grown under LD conditions; PRJNA730091: Effects of annual AmMAR1/AmMAR2 on gene expression; PRJNA730701: Gene regulation underlying the vernalization response in annual and perennial plants; PRJNA731145: Whole-genome sequencing of *A. montbretiana* Boiss; MZ736051-MZ736067: chr8 MAF region PacBio assembly). All study data are included in the article and/or *SI Appendix*. There are no additional data underlying this work.

## References

[1] M. C. Albani, G. Coupland, Comparative Analysis of Flowering in Annual and Perennial Plants. Curr. Top. Dev. Biol., Plant Development, M. C. P. Timmermans, Ed. (Academic Press, 2010), vol. 91, pp. 323–348. 10.1016/S0070-2153(10)91011-9 20705187

[2] J. Friedman, The evolution of annual and perennial plant life histories: Ecological correlates and genetic mechanisms. Annu. Rev. Ecol. Evol. Syst. 51, 461–481 (2020).

[3] S. C. Barrett, The evolution of plant sexual diversity. Nat. Rev. Genet. 3, 274–284 (2002). 11967552 10.1038/nrg776

[4] F. Y. Hu., Convergent evolution of perenniality in rice and sorghum. Proc. Natl. Acad. Sci. U.S.A. 100, 4050–4054 (2003). 12642667 10.1073/pnas.0630531100 PMC153046

[5] C. Kiefer., Divergence of annual and perennial species in the Brassicaceae and the contribution of cis-acting variation at FLC orthologues. Mol. Ecol. 26, 3437–3457 (2017). 28261921 10.1111/mec.14084 PMC5485006

[6] G. Stebbins, Flowering Plants: Evolution Above the Species Level (Belknap Press of Harvard Univeristy Press, Cambridge, 1974).

[7] D. B. Lowry, J. H. Willis, A widespread chromosomal inversion polymorphism contributes to a major life-history transition, local adaptation, and reproductive isolation. PLoS Biol. 8, e1000500 (2010). 20927411 10.1371/journal.pbio.1000500 PMC2946948

[8] M. A. Grillo., Genetic architecture for the adaptive origin of annual wild rice, *oryza nivara*. Evolution 63, 870–883 (2009). 19236476 10.1111/j.1558-5646.2008.00602.x

[9] D. B. Lowry., The case for the continued use of the genus name *Mimulus* for all monkeyflowers. Taxon 68, 617–623 (2019).

[10] D. B. Lowry, R. C. Rockwood, J. H. Willis, Ecological reproductive isolation of coast and inland races of *Mimulus guttatus*. Evolution 62, 2196–2214 (2008). 18637837 10.1111/j.1558-5646.2008.00457.x PMC11110535

[11] Y. Hyun., A regulatory circuit conferring varied flowering response to cold in annual and perennial plants. Science 363, 409–412 (2019). 30679374 10.1126/science.aau8197

[12] R. Wang., *PEP1* regulates perennial flowering in *Arabis alpina*. Nature 459, 423–427 (2009). 19369938 10.1038/nature07988

[13] S. B. Carroll, Evolution at two levels: On genes and form. PLoS Biol. 3, e245 (2005). 16000021 10.1371/journal.pbio.0030245 PMC1174822

[14] N. Castelán-Muñoz., MADS-box genes are key components of genetic regulatory networks involved in abiotic stress and plastic developmental responses in plants. Front. Plant Sci. 10, 853 (2019). 31354752 10.3389/fpls.2019.00853 PMC6636334

[15] T. T. Hu., The *Arabidopsis lyrata* genome sequence and the basis of rapid genome size change. Nat. Genet. 43, 476–481 (2011). 21478890 10.1038/ng.807 PMC3083492

[16] W. B. Jiao., Improving and correcting the contiguity of long-read genome assemblies of three plant species using optical mapping and chromosome conformation capture data. Genome Res. 27, 778–786 (2017). 28159771 10.1101/gr.213652.116 PMC5411772

[17] E. M. Willing., Genome expansion of *Arabis alpina* linked with retrotransposition and reduced symmetric DNA methylation. Nat. Plants 1, 1–7 (2015). 10.1038/nplants.2014.23 27246759

[18] R. Karl, C. Kiefer, S. W. Ansell, M. A. Koch, Systematics and evolution of Arctic-Alpine *Arabis alpina* (Brassicaceae) and its closest relatives in the eastern Mediterranean. Am. J. Bot. 99, 778–794 (2012). 22454383 10.3732/ajb.1100447

[19] S. D. Michaels, R. M. Amasino, *FLOWERING LOCUS C* encodes a novel *MADS* domain protein that acts as a repressor of flowering. Plant Cell 11, 949–956 (1999). 10330478 10.1105/tpc.11.5.949 PMC144226

[20] C. C. Sheldon., The *FLF* MADS box gene: A repressor of flowering in Arabidopsis regulated by vernalization and methylation. Plant Cell 11, 445–458 (1999). 10072403 10.1105/tpc.11.3.445 PMC144185

[21] S. Bergonzi., Mechanisms of age-dependent response to winter temperature in perennial flowering of *Arabis alpina*. Science 340, 1094–1097 (2013). 23723236 10.1126/science.1234116

[22] A. Lazaro, E. Obeng-Hinneh, M. C. Albani, Extended vernalization regulates inflorescence fate in *Arabis alpina* by stably silencing *PERPETUAL FLOWERING1*. Plant Physiol. 176, 2819–2833 (2018). 29467177 10.1104/pp.17.01754 PMC5884582

[23] R. Finkers., The construction of a *Solanum habrochaites* LYC4 introgression line population and the identification of QTLs for resistance to *Botrytis cinerea*. Theor. Appl. Genet. 114, 1071–1080 (2007). 17273845 10.1007/s00122-006-0500-2 PMC1913174

[24] M. Yano., *Hd1*, a major photoperiod sensitivity quantitative trait locus in rice, is closely related to the Arabidopsis flowering time gene *CONSTANS*. Plant Cell 12, 2473–2484 (2000). 11148291 10.1105/tpc.12.12.2473 PMC102231

[25] X. Gu., Arabidopsis FLC clade members form flowering-repressor complexes coordinating responses to endogenous and environmental cues. Nat. Commun. 4, 1–10 (2013). 10.1038/ncomms2947 PMC3709509 23770815

[26] O. J. Ratcliffe, R. W. Kumimoto, B. J. Wong, J. L. Riechmann, Analysis of the Arabidopsis *MADS AFFECTING FLOWERING* gene family: *MAF2* prevents vernalization by short periods of cold. Plant Cell 15, 1159–1169 (2003). 12724541 10.1105/tpc.009506 PMC153723

[27] G. Theißen, F. Rümpler, L. Gramzow, Array of MADS-box genes: Facilitator for rapid adaptation? Trends Plant Sci. 23, 563–576 (2018). 29802068 10.1016/j.tplants.2018.04.008

[28] Z. Yang, PAML 4: Phylogenetic analysis by maximum likelihood. Mol. Biol. Evol. 24, 1586–1591 (2007). 17483113 10.1093/molbev/msm088

[29] X. Lai, H. Daher, A. Galien, V. Hugouvieux, C. Zubieta, Structural basis for plant MADS transcription factor oligomerization. Comput. Struct. Biotechnol. J. 17, 946–953 (2019). 31360333 10.1016/j.csbj.2019.06.014 PMC6639411

[30] V. Tilmes., Gibberellins act downstream of *Arabis* PERPETUAL FLOWERING1 to accelerate floral induction during vernalization. Plant Physiol. 180, 1549–1563 (2019). 31097676 10.1104/pp.19.00021 PMC6752923

[31] T. Kania, D. Russenberger, S. Peng, K. Apel, S. Melzer, FPF1 promotes flowering in Arabidopsis. Plant Cell 9, 1327–1338 (1997). 9286110 10.1105/tpc.9.8.1327 PMC157001

[32] C. A. Airoldi, M. McKay, B. Davies, *MAF2* is regulated by temperature-dependent splicing and represses flowering at low temperatures in parallel with *FLM*. PLoS One 10, e0126516 (2015). 25955034 10.1371/journal.pone.0126516 PMC4425511

[33] N. Panchy, M. Lehti-Shiu, S. H. Shiu, Evolution of gene duplication in plants. Plant Physiol. 171, 2294–2316 (2016). 27288366 10.1104/pp.16.00523 PMC4972278

[34] Y. Van de Peer, J. A. Fawcett, S. Proost, L. Sterck, K. Vandepoele, The flowering world: A tale of duplications. Trends Plant Sci. 14, 680–688 (2009). 19818673 10.1016/j.tplants.2009.09.001

[35] Y. Wang, X. Wang, A. H. Paterson, Genome and gene duplications and gene expression divergence: A view from plants. Ann. N. Y. Acad. Sci. 1256, 1–14 (2012). 22257007 10.1111/j.1749-6632.2011.06384.x

[36] A. L. Caicedo, C. Richards, I. M. Ehrenreich, M. D. Purugganan, Complex rearrangements lead to novel chimeric gene fusion polymorphisms at the *Arabidopsis thaliana MAF2-5* flowering time gene cluster. Mol. Biol. Evol. 26, 699–711 (2009). 19139056 10.1093/molbev/msn300

[37] S. M. Rosloski, S. S. Jali, S. Balasubramanian, D. Weigel, V. Grbic, Natural diversity in flowering responses of *Arabidopsis thaliana* caused by variation in a tandem gene array. Genetics 186, 263–276 (2010). 20551443 10.1534/genetics.110.116392 PMC2940291

[38] N. Hohmann, E. M. Wolf, M. A. Lysak, M. A. Koch, A time-calibrated road map of Brassicaceae species radiation and evolutionary history. Plant Cell 27, 2770–2784 (2015). 26410304 10.1105/tpc.15.00482 PMC4682323

[39] L. A. Nikolov., Resolving the backbone of the Brassicaceae phylogeny for investigating trait diversity. New Phytol. 222, 1638–1651 (2019). 30735246 10.1111/nph.15732

[40] H. Li., Genome-wide identification of flowering-time genes in *Brassica* species and reveals a correlation between selective pressure and expression patterns of vernalization-pathway genes in *Brassica napus*. Int. J. Mol. Sci. 19, 3632 (2018). 10.3390/ijms19113632 PMC6274771 30453667

[41] K. C. Scortecci, S. D. Michaels, R. M. Amasino, Identification of a MADS-box gene, *FLOWERING LOCUS M*, that represses flowering. Plant J. 26, 229–236 (2001). 11389763 10.1046/j.1365-313x.2001.01024.x

[42] J. D. Werner., Quantitative trait locus mapping and DNA array hybridization identify an *FLM* deletion as a cause for natural flowering-time variation. Proc. Natl. Acad. Sci. U.S.A. 102, 2460–2465 (2005). 15695584 10.1073/pnas.0409474102 PMC548991

[43] S. DeBolt, Copy number variation shapes genome diversity in Arabidopsis over immediate family generational scales. Genome Biol. Evol. 2, 441–453 (2010). 20624746 10.1093/gbe/evq033 PMC2997553

[44] K. Hanada, C. Zou, M. D. Lehti-Shiu, K. Shinozaki, S. H. Shiu, Importance of lineage-specific expansion of plant tandem duplicates in the adaptive response to environmental stimuli. Plant Physiol. 148, 993–1003 (2008). 18715958 10.1104/pp.108.122457 PMC2556807

[45] S. Maere., Modeling gene and genome duplications in eukaryotes. Proc. Natl. Acad. Sci. U.S.A. 102, 5454–5459 (2005). 15800040 10.1073/pnas.0501102102 PMC556253

[46] S. Maere, Y. Van de Peer, “Duplicate retention after small- and large-scale duplications” in Evolution after Gene Duplication, K. Dittmar, D. Liberles, Eds. (John Wiley & Sons, 2010), pp. 31–56.

[47] G. Capovilla, E. Symeonidi, R. Wu, M. Schmid, Contribution of major FLM isoforms to temperature-dependent flowering in *Arabidopsis thaliana*. J. Exp. Bot. 68, 5117–5127 (2017). 29036339 10.1093/jxb/erx328 PMC5853260

[48] S. Sureshkumar, C. Dent, A. Seleznev, C. Tasset, S. Balasubramanian, Nonsense-mediated mRNA decay modulates *FLM-dependent* thermosensory flowering response in Arabidopsis. Nat. Plants 2, 1–7 (2016). 10.1038/nplants.2016.55 27243649

[49] T. Casneuf, S. De Bodt, J. Raes, S. Maere, Y. Van de Peer, Nonrandom divergence of gene expression following gene and genome duplications in the flowering plant *Arabidopsis thaliana*. Genome Biol. 7, 1–13 (2006). 10.1186/gb-2006-7-2-r13 PMC1431724 16507168

[50] Li Z., Expression pattern divergence of duplicated genes in rice. BMC Bioinformatics 10 (suppl 6), S8 (2009). 10.1186/1471-2105-10-S6-S8 PMC2697655 19534757

[51] A. Kinoshita, R. Richter, Genetic and molecular basis of floral induction in *Arabidopsis thaliana*. J. Exp. Bot. 71, 2490–2504 (2020). 32067033 10.1093/jxb/eraa057 PMC7210760

[52] E. Madrid, J. W. Chandler, G. Coupland, Gene regulatory networks controlled by FLOWERING LOCUS C that confer variation in seasonal flowering and life history. J. Exp. Bot. 72, 4–14 (2021). 32369593 10.1093/jxb/eraa216 PMC7816851

[53] V. Coustham., Quantitative modulation of polycomb silencing underlies natural variation in vernalization. Science 337, 584–587 (2012). 22798408 10.1126/science.1221881

[54] H. Li, R. Durbin, Fast and accurate short read alignment with Burrows-Wheeler transform. Bioinformatics 25, 1754–1760 (2009). 19451168 10.1093/bioinformatics/btp324 PMC2705234

[55] S. F. Altschul, W. Gish, W. Miller, E. W. Myers, D. J. Lipman, Basic local alignment search tool. J. Mol. Biol. 215, 403–410 (1990). 2231712 10.1016/S0022-2836(05)80360-2

[56] B. J. Haas, A. L. Delcher, J. R. Wortman, S. L. Salzberg, DAGchainer: A tool for mining segmental genome duplications and synteny. Bioinformatics 20, 3643–3646 (2004). 15247098 10.1093/bioinformatics/bth397

[57] L. Stephan, V. Tilmes, M. Hülskamp, Selection and validation of reference genes for quantitative Real-Time PCR in *Arabis alpina*. PLoS One 14, e0211172 (2019). 30830921 10.1371/journal.pone.0211172 PMC6398851

[58] R. Patro, G. Duggal, M. I. Love, R. A. Irizarry, C. Kingsford, Salmon provides fast and bias-aware quantification of transcript expression. Nat. Methods 14, 417–419 (2017). 28263959 10.1038/nmeth.4197 PMC5600148

[59] J. T. Leek, W. E. Johnson, H. S. Parker, A. E. Jaffe, J. D. Storey, The sva package for removing batch effects and other unwanted variation in high-throughput experiments. Bioinformatics 28, 882–883 (2012). 22257669 10.1093/bioinformatics/bts034 PMC3307112

[60] M. I. Love, W. Huber, S. Anders, Moderated estimation of fold change and dispersion for RNA-seq data with DESeq2. Genome Biol. 15, 1–21 (2014). 10.1186/s13059-014-0550-8 PMC4302049 25516281

[61] S. M. Kiełbasa, R. Wan, K. Sato, P. Horton, M. C. Frith, Adaptive seeds tame genomic sequence comparison. Genome Res. 21, 487–493 (2011). 21209072 10.1101/gr.113985.110 PMC3044862

[62] M. Goel, H. Sun, W.-B. Jiao, K. Schneeberger, SyRI: Finding genomic rearrangements and local sequence differences from whole-genome assemblies. Genome Biol. 20, 1–13 (2019). 31842948 10.1186/s13059-019-1911-0 PMC6913012

[63] S. Capella-Gutiérrez, J. M. Silla-Martínez, T. Gabaldón, trimAl: A tool for automated alignment trimming in large-scale phylogenetic analyses. Bioinformatics 25, 1972–1973 (2009). 19505945 10.1093/bioinformatics/btp348 PMC2712344

[64] R. C. Edgar, MUSCLE: Multiple sequence alignment with high accuracy and high throughput. Nucleic Acids Res. 32, 1792–1797 (2004). 15034147 10.1093/nar/gkh340 PMC390337

[65] D. Posada, jModelTest: Phylogenetic model averaging. Mol. Biol. Evol. 25, 1253–1256 (2008). 18397919 10.1093/molbev/msn083

[66] S. Guindon, F. Delsuc, J. F. Dufayard, O. Gascuel, Estimating maximum likelihood phylogenies with PhyML. Methods Mol. Biol. 537, 113–137 (2009). 19378142 10.1007/978-1-59745-251-9_6

[67] J. Huerta-Cepas, F. Serra, P. Bork, ETE 3: Reconstruction, analysis, and visualization of phylogenomic data. Mol. Biol. Evol. 33, 1635–1638 (2016). 26921390 10.1093/molbev/msw046 PMC4868116

