# Supplementary material for: Transposition and duplication of MADS-domain transcription factor genes in annual and perennial Arabis species modulates flowering
Source: Proc Natl Acad Sci U S A. 2021 Sep 21;118(39):e2109204118. doi: 10.1073/pnas.2109204118 (PMC8488671; doi:10.1073/pnas.2109204118)
Supplement: Supplementary File [file pnas.2109204118.sapp.pdf]

PNAS
www.pnas.org

Supplementary Information for

Duplication and transposition of MADS-domain transcription factor genes in annual and perennial *Arabis* species modulates flowering

Eva Madrid[1], Edouard Severing[1], Elisa de Ansorena[1], Christiane Kiefer[1,2], Luise Brand[1], Rafael Martinez-Gallegos[1,2], Stefan Woetzel[1,3,4], Ulla Kemi[1], Wen-Biao Jiao[1], Korbinian Schneeberger[1] and George Coupland[1,*]

George Coupland
Email: coupland@mpipz.mpg.de

This PDF file includes:

Supplementary text
Figures S1 to S6 (not allowed for Brief Reports)
Legends for Datasets S1 to S6
SI References

Extended materials and methods

Plant material and sequence availability

A. nova subsp. *iberica* was provided by Santiago Martin Bravo, University of Sevilla, Spain, while *Arabis auriculata (*B-2006-0564) and *A. nordmanniana* (RK219) were provided by MA Koch (1). In addition, five other accessions of *A. alpina* collected across a wide geographical range were used: two from Scandinavia (S4a and S4b), two from France (Pic and Gal) and *A. alpina* from Tanzania (accession number 109656, provided by Heidelberg University Botanical Garden).

The *A. alpina* reference genome used in this manuscript was version 5.1(http://www.arabis-alpina.org/refseq.html). The genomes of *A. nova* subsp. *iberica* , *A. auriculata* and the five *A. alpina* accessions described below were *de novo* assembled from PacBio reads using Canu (2). The available *A. nordmanniana* genome partially assembled under BioProject PRJNA258061 was used.

The *A. montbretiana* genome sequence (LNCH02000000; BioSample SAMN02983095) was assembled using ALLPATHS-LG with default parameters using Illumina sequencing reads (3). The assembled scaffolds were blasted against the *A. thaliana* mitochondrial (The Arabidopsis Genome Initiative 2000), the *A. alpina* chloroplast (4) and the NCBI bacterial nucleotide genome sequences, to identify scaffolds assembled from the organelle genome sequences and to remove contaminant scaffolds. Scaffolds were anchored to chromosomes using *A. alpina* reference sequence version (5). Gene annotation was performed by integrating evidence from ab initio prediction, RNA-seq paired-end reads and homologous protein sequence alignments.

MAR sequences on chromosome 2 and MAF cluster sequences on chromosome 8 used in this study have been deposited on NCBI under accession numbers MZ736051 to MZ736067.

Plasmid construction and plant transformation

To generate *pAmMAR1::gAmMAR1* and *pAmMAR2::gAmMAR2* transgenic lines, sequences upstream from the translational start site and downstream to the next annotated gene for both genes were amplified by PCR from genomic DNA and ligated to the linear vector pSTB205 vector by PIPE cloning (6). The sequences of the primers used to generate cloned fragments of both genes are provided in Dataset S1. The DNA

constructs were then recombined using GATEWAY into the pEarleyGate301 (7) binary vector and transformed into *pep1-1* using the *Agrobacterium*-mediated method (8).

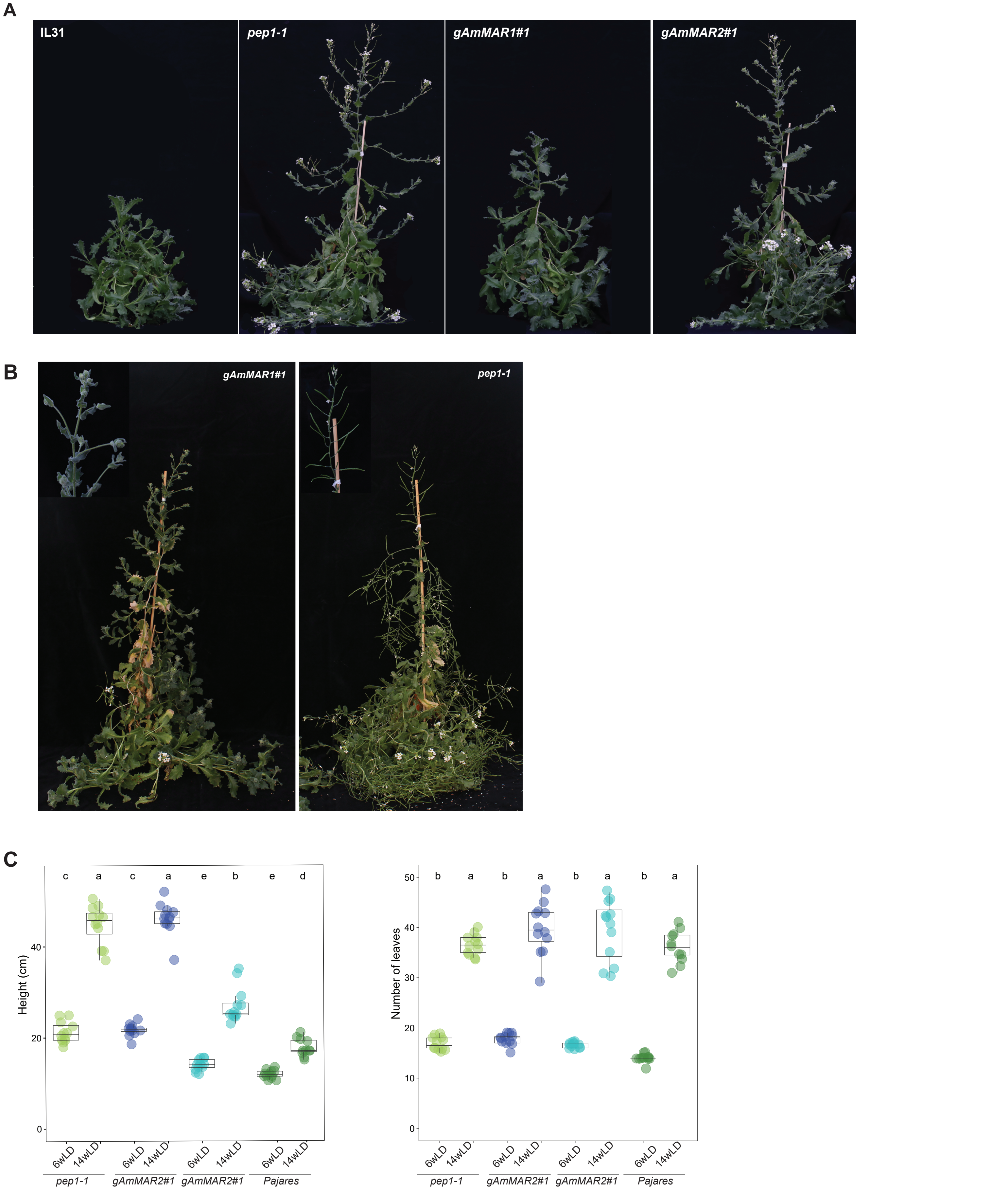

Figure S1. (A) plants grown for 10 weeks illustrate different flowering times. (B) Plants of *pAmMAR1::gAmMAR1* (left) and *pep1-1* (right) grown for 14 weeks in long days, showing the flowering behavior of the main and lateral shoots. *pAmMAR1::gAmMAR1* plant (left) flowers from the secondary shoots, whereas flowering in the main shoot is repressed. *pep1-1* (right) flowers from primary and secondary shoots. (C) Boxplots showing plant height and leaf number 6 and 14 weeks after germination in long days. Height, but not leaf number, of late-flowering *pAmMAR1::g-MAR1* plants and the reference accession, *A. alpina* Pajares, is reduced compared to *pep1-1.*

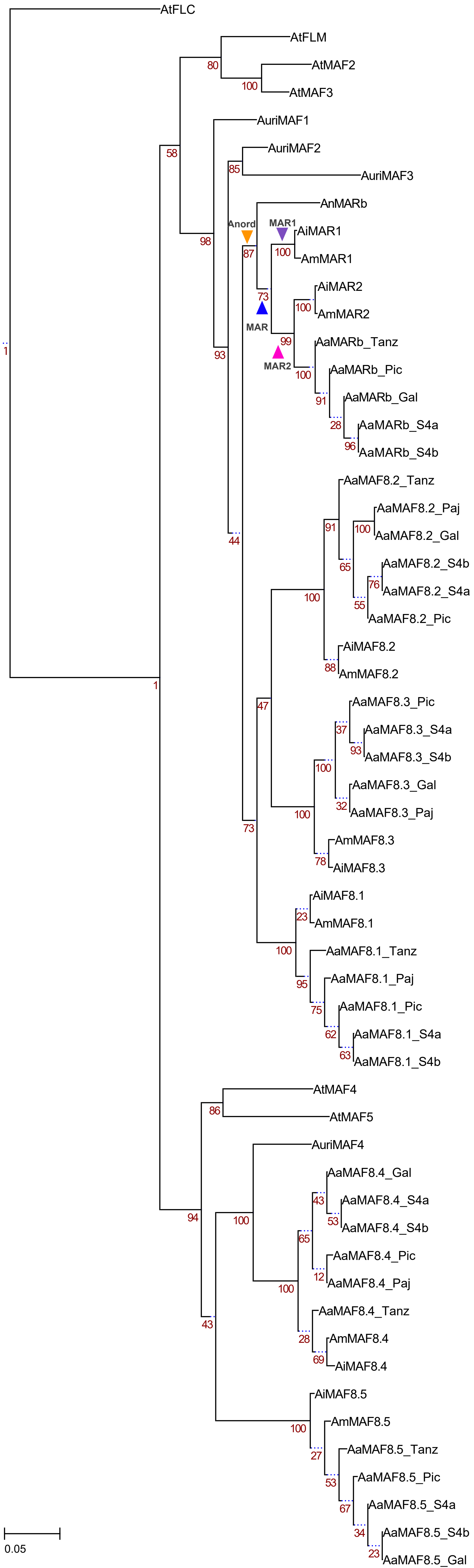

Figure S2. Phylogenetic tree used for dN/dS analysis. Maximum likelihood tree used for the dN/dS analysis using PAML. Node values indicate bootstrap support. Colored triangles denote the branches that were targeted in the branch- and branch-site tests for positive selection.

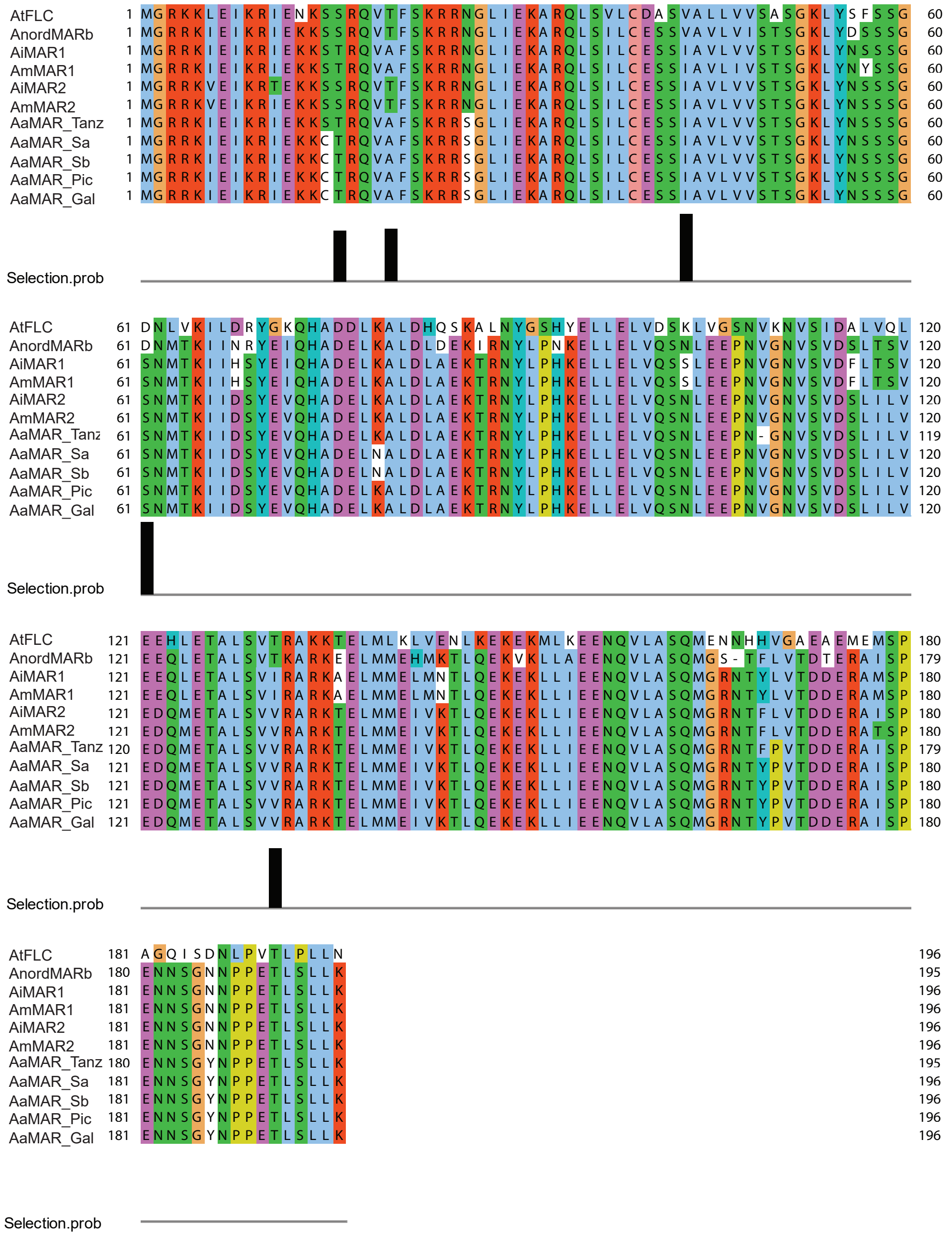

Figure S3. Scores along the alignment used for dN/dS. Alignment of the protein sequences of the MAR group including A. nordmanniana, and AtFLC as the outgroup used by PAML as the reference for alignment. The bar represented at the bottom indicates the sites with probability scores 0.7 and above.

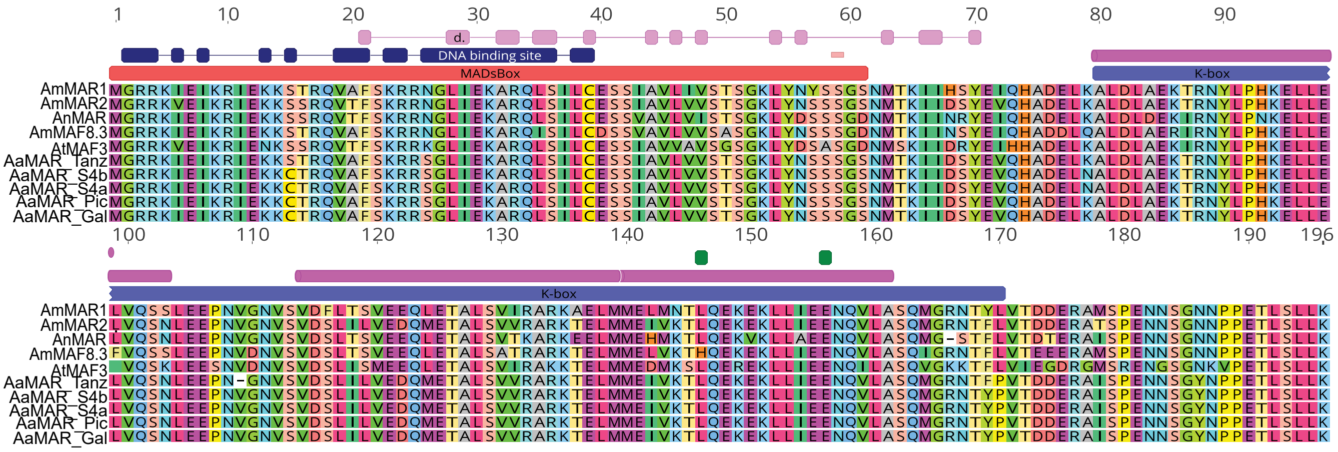

Figure S4. Sequence comparisons of the predicted MAR proteins from different Arabis accessions. The MADS domain (red) and K domain (lilac) are represented, as well as the DNA-binding domain (blue), DNA-interaction domain (pink), and alpha helices (purple). AtMAF3 and one of its orthologues in *A. montbretiana* (AmMAF8.3) are also included.

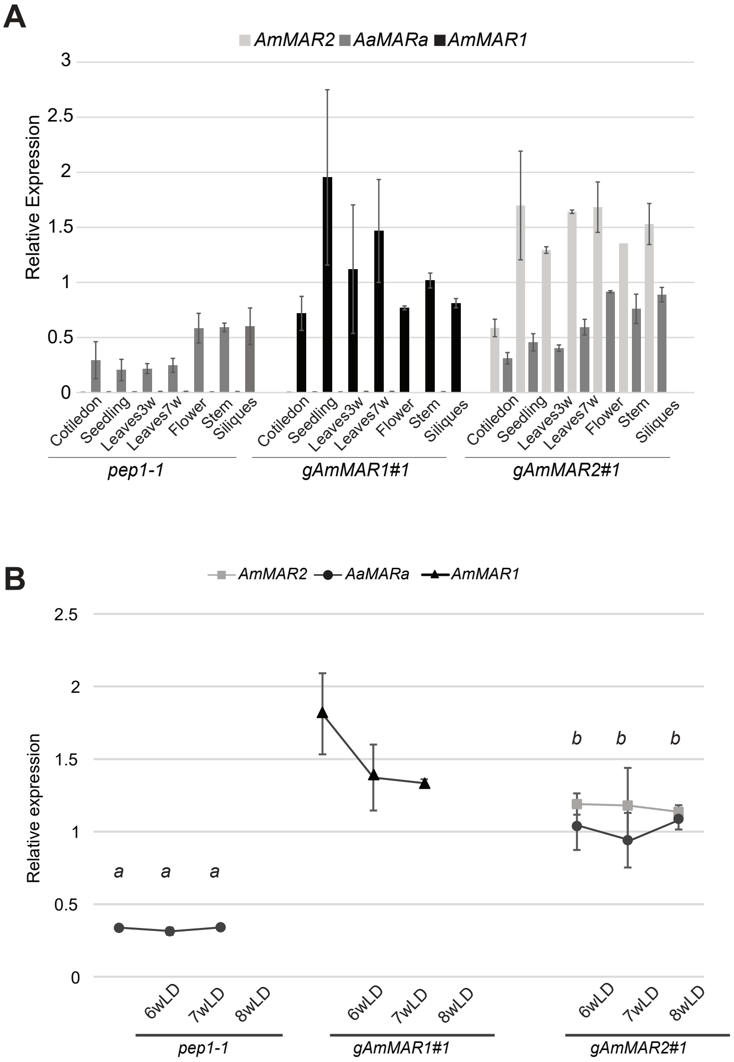

Figure S5. Level of *AaMARa*, *AmMAR1* and *AmMAR2* mRNA in (A) different tissues/genotypes and (B) in apices of *pAmMAR1:gAmMAR1* and *pAmMAR2:gAmMAR2* transgenic lines and *pep1-1* at shown number of weeks after germination.

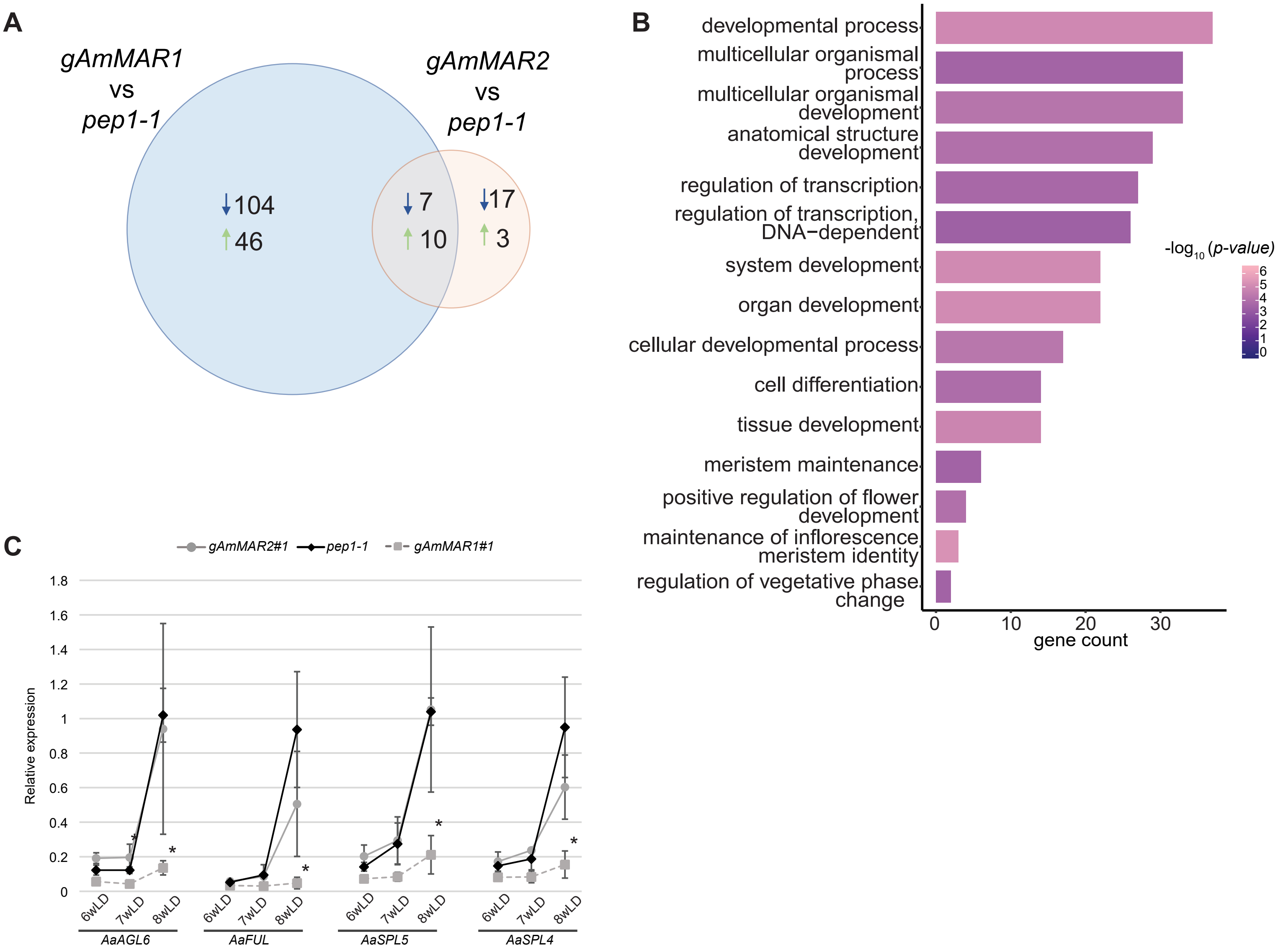

Figure S6. RNAseq analysis of 6 weeks old plants. (A) Venn diagram representing the overlap of differentially expressed genes (DEGs) in *pAmMAR1::gAmMAR1* or *pAmMAR2::gAmMAR2* transgenic lines compared with pep1-1. Up- and downregulated genes are represented by green and blue arrows, respectively. (B) GO-enrichment analysis of the genes differentially expressed in *pAmMAR1::gAmMAR1* compared with *pep1-1*. Categories were ordered according to the gene counts they represented. The color code indicates the -$\log_{10}$(*p-value*). (C) Levels of *AaAGL6*, *AaFUL*, *AaSPL5* and *AaSPL4 mRNA* in lateral inflorescence shoot apices of *pAmMAR1:gAmMAR1* and *pAmMAR2:gAmMAR2* transgenic lines and *pep1-1* at shown number of weeks after germination. Transcripts are differentially expressed in lateral inflorescence shoots at shown number of weeks after germination. The first four lateral shoots from the base of the plants were used. Letters and asterisks indicate significant differences determined by multiple pairwise comparisons using Tukey's HSD test ($p \leq 0.05$).Transcriptome analysis identifies flowering genes repressed in *gAmMAR1#1* transgenic plants.

Dataset S1 (separate file). A list of primers used in this study.

Dataset S2 (separate file). Whole-genome sequencing results of introgression lines (IL). Coordinates of introgressions of *A. montbretiana* (Am) in *A. alpina* (Aa) are given, as well as the number of genes present in each fragment. The fpkm were calculated against the genome and are indicated in column D, as well as the genotype inferred from this mapping (K and L). NA, not applicable: No orthologue of the gene is present in the other species.

Dataset S3 (separate file). PAML results for the branch model. Model 1 is the model for which two separate dN/dS ratios were sought: One for the target branch (test) and the other for the rest of the tree (background). Model 2 is the model in which a single ratio was calculated for the whole tree. The table also provides the likelihoods (lnL), number of parameters (np) for both models, the values for the likelihood ratio test (2*(lnL1 - lnL2), degrees of freedom: np1 - np2) and the resulting *p*-value. * Model 2 also has two ratios, but the target branch ratio is fixed at 1.

Dataset S4 (separate file). PAML results for the branch-site model test of positive selection (Model1) and neutrality (Model2). The for - and background dn/ds for four site classes are provided, including the likelihoods (lnL) for each model, the number of parameters (np) and the values used for the likelihood ratio test (2*(lnL1 - lnL2), degrees of freedom: np1 - np2).

Dataset S5 (separate file). Transcripts identified as being differentially expressed between *pAmMAR1::gMAR1 or pAmMAR2::gMAR2* and *pep1-1*.

Dataset S6 (separate file). DESeq2 analysis for *MAF* genes during a vernalization time course in *A. thaliana*, *A. montbretiana*, *A. alpina* Pajares and *pep1-1*. Log_2 fold change was calculated with reference to expression at the first time point, before plants were vernalized.

SI References

1. Kiefer M, *et al.* (2014) BrassiBase: introduction to a novel knowledge database on Brassicaceae evolution. *Plant Cell Physiol* 55(1):e3.
2. Koren S, *et al.* (2017) Canu: scalable and accurate long-read assembly via adaptive k-mer weighting and repeat separation. *Genome Res.* 27(5):722-736.
3. Gnerre S, *et al.* (2011) High-quality draft assemblies of mammalian genomes from massively parallel sequence data. *Proc Natl Acad Sci USA* 108(4):1513.
4. Melodelima C & Lobréaux S (2013) Complete *Arabis alpina* chloroplast genome sequence and insight into its polymorphism. *Meta gene* 1:65-75.
5. Willing EM, *et al.* (2015) Genome expansion of *Arabis alpina* linked with retrotransposition and reduced symmetric DNA methylation. *Nat Plants* 1:14023.
6. Klock HE & Lesley SA (2009) The Polymerase Incomplete Primer Extension (PIPE) method applied to high-throughput cloning and site-directed mutagenesis. *Methods Mol Biol* 498:91-103.
7. Earley KW, *et al.* (2006) Gateway-compatible vectors for plant functional genomics and proteomics. *Plant J.* 45(4):616-629.
8. Wang R, *et al.* (2009) *PEP1* regulates perennial flowering in *Arabis alpina*. *Nature* 459(7245):423-427.
